# Multiple roles of *Pseudomonas aeruginosa* TBCF10839 PilY1 in motility, transport and infection

**DOI:** 10.1111/j.1365-2958.2008.06559.x

**Published:** 2008-12-10

**Authors:** Yu-Sing Tammy Bohn, Gudrun Brandes, Elza Rakhimova, Sonja Horatzek, Prabhakar Salunkhe, Antje Munder, Andrea van Barneveld, Doris Jordan, Florian Bredenbruch, Susanne Häußler, Kathrin Riedel, Leo Eberl, Peter Østrup Jensen, Thomas Bjarnsholt, Claus Moser, Niels Hoiby, Burkhard Tümmler, Lutz Wiehlmann

**Affiliations:** 1 Klinische ForschergruppeOE 6710; 2 Abteilung ZellbiologieOE 4130 and Betriebseinheit Elektronenmikroskopie-LaborOE 8840; 3 Abteilung für Medizinische Mikrobiologie und KrankenhaushygieneOE 5210, Medizinische Hochschule Hannover, Carl-Neuberg-Str. 1, D-30625 Hannover, Germany; 4 Abteilung für Zellbiologie und Immunologie, Helmholtz Institut für InfektionsforschungInhoffenstr. 7, D-38124 Braunschweig, Germany; 5 Abteilung für Mikrobiologie, Institut für Pflanzenbiologie, Universität ZürichWinterthurerstrasse 190, CH-8057 Zürich, Switzerland; 6 Department of Clinical Microbiology, Rigshospitalet, afsnit 9301, Department of Clinical MicrobiologyJuliane Maries Vej 22, DK-2100 Copenhagen, Denmark; 7 BioScience and Technology BioCentrum-DTU, Building 227, Technical University of DenmarkDK-2800 Lyngby, Denmark

## Abstract

Polymorphonuclear neutrophils are the most important mammalian host defence cells against infections with *Pseudomonas aeruginosa.* Screening of a signature tagged mutagenesis library of the non-piliated *P. aeruginosa* strain TBCF10839 uncovered that transposon inactivation of its *pilY1* gene rendered the bacterium more resistant against killing by neutrophils than the wild type and any other of the more than 3000 tested mutants. Inactivation of *pilY1* led to the loss of twitching motility in twitching-proficient wild-type PA14 and PAO1 strains, predisposed to autolysis and impaired the secretion of quinolones and pyocyanin, but on the other hand promoted growth in stationary phase and bacterial survival in murine airway infection models. The PilY1 population consisted of a major full-length and a minor shorter PilY1* isoform. PilY1* was detectable in small extracellular quinolone-positive aggregates, but not in the pilus. *P. aeruginosa* PilY1 is not an adhesin on the pilus tip, but assists in pilus biogenesis, twitching motility, secretion of secondary metabolites and in the control of cell density in the bacterial population.

## Introduction

*Pseudomonas aeruginosa* is a metabolically versatile Gram-negative bacterium, which inhabits terrestrial and aquatic as well as animal, human and plant host associated environments (Ramos, 2004). This opportunistic pathogen is the most dominant bacterium causing chronic infections in the cystic fibrosis (CF) lung (Lyczak *et al*., 2002; Gómez and Prince, 2007) and has emerged as an important causative agent of nosocomial infections, particularly in intensive care units (Trautmann *et al*., 2005). The first step in establishing an infection is the adherence and colonization of the epithelium, which is mediated, in part, by type IV pili (T4P) (Klausen *et al*., 2003; Giltner *et al*., 2006). These T4P are also receptors for bacteriophages and mediate a mode of surface translocation termed twitching motility (Mattick, 2002).

*Pseudomonas aeruginosa* T4P are polar organelles that are composed of a single protein subunit, PilA. The PilA subunits are first synthesized as prepilins. In the course of assembly in the periplasmic space, the leader peptide is cleaved and the resulting N-terminal residue is methylated (Whitchurch, 2006). The assembled pilus filament is extruded through the outer membrane via a gated channel, the multimeric PilQ secretin (Martin *et al*., 1993; Wolfgang *et al*., 2000; Collins *et al*., 2001). Besides the major T4P prepilin PilA, the *P. aeruginosa* genome contains one gene cluster (PA4549–PA4556) that encodes PilY1, PilY2 and the six minor T4P prepilins FimT, FimU, PilV, PilW, PilX and PilE (Mattick, 2002; Whitchurch, 2006). The *fimU-pilVWXY1Y2E* genes are cotranscribed in an operon which is under the control of the response regulator AlgR (Belete *et al*., 2008).

The minor prepilins possess the canonical hydrophobic N-terminal sequence signature of prepilins (Mattick, 2002; Whitchurch, 2006). They are membrane-located and are required for pilus biogenesis (Russell and Darzins, 1994; Alm *et al*., 1996), but do not seem to be incorporated into the extracellular T4P structure. The two non-prepilin proteins that are encoded by the operon *fimU-pilVWXY1Y2E,* PilY1 (PA4554) and PilY2 (PA4555) are also necessary for pilus formation (Alm *et al*., 1996). In addition, PilY1 has been reported to be involved in the control of the expression of the *P. aeruginosa* lipase LipC (PA4813) (Martínez *et al*., 1999). The PilY1 protein carries a prepilin peptidase export signal and is transported across the inner membrane (Lewenza *et al*., 2005). PilY1 of *P. aeruginosa* PAO1 shares 43% homology to the C-terminal sequence of PilC2 of *Neisseria meningitidis* and of *Neisseria gonorrhoeae*. Neisserial PilC resides in the bacterial outer membrane (Rahman *et al*., 1997) and in the pilus tip (Rudel *et al*., 1995). The PilC proteins whose expression can be switched on and off by phase variation (Rytkönen *et al*., 2004), are major adhesins and regulate PilT-mediated T4P retraction in *Neisseria* (Morand *et al*., 2004). This datum indicates that the 3.5 kb *P. aeruginosa pilY1* homologue may encode a protein with multiple functions including, but not restricted to, pilus biogenesis.

*Pseudomonas aeruginosa* TBCF10839 (Tümmler *et al.*, 1991) is a non-piliated CF isolate that carries an out-of-frame deletion in the *pilQ* gene (Chang *et al*., 2007). Hence this strain provided the opportunity to explore functions of pilus biogenesis genes that are not directly related to their essential roles in piliation. In this study the strain TBCF10839 and mutants thereof were exploited to gain knowledge about the yet unresolved function(s) of *P. aeruginosa* PilY1. Besides its known roles in piliation and twitching, PilY1 was found to be involved in numerous other phenotypes, namely the secretion of phenazines and quinolines, bacterial cell lysis, virulence and mammalian host defence.

## Results

### Screening of a signature tagged mutagenesis library of *P. aeruginosa* TBCF10839 in a polymorphonuclear neutrophil phagocytosis assay

Polymorphonuclear neutrophils (PMNs) are the most important mammalian host defence cells against infections with *P. aeruginosa* (Döring *et al*., 1995). A mini-Tn*5* plasposon library of the CF airway isolate TBCF10839 carrying variable V_40_ (V = A, G, C) oligonucleotide signature tags were screened for survival in freshly prepared human PMNs in phagocytosis assays. Batches of 48 mutants each carrying a different signature tag were exposed at a total multiplicity of infection of 10 to PMNs and viable bacteria were recovered after 2 h. Numerous transposon mutants were identified which survived at significant lower frequencies in the phagocytosis assays than the wild-type strain (L. Wiehlmann, in preparation). Unexpectedly, four mutants consistently showed higher survival rates than TBCF10839, suggesting that the inactivation of this gene by the insertion of a transposon increased resistance to killing by PMNs. Sequencing revealed that the transposon had inserted into two genes of yet unknown function (P. Salunkhe, in preparation) or into two different positions of the 3486 bp large pilus biogenesis gene *pilY1*[PA4554, positions 1617 (mutant 10CB5) or 2308 (mutant 25C8)].

### PilY1 phenotype in a non-piliated host strain

PilY1 was identified in *P. aeruginosa* as the homologue of the gonococcal PilC which is the T4P tip-located adhesin (Alm *et al*., 1996). *P. aeruginosa* strain TBCF10839, however, is non-piliated due to an out-of-frame deletion in the *pilQ* gene (Chang *et al*., 2007). The dodecameric transmembrane protein PilQ plays a central role in extruding the pilus fibre through the bacterial outer membrane and thereby facilitates the presence and functionality of extracellular pili (Whitchurch, 2006). Hence, the transposon mutants in the *pilY1* gene of strain TBCF10839 are double knockouts in the polycistronic operons *pilMNOPQ* (PA5044–PA5040) and *pilVWXY1Y2E* (PA4551–PA4556). Correspondingly, neither strain TBCF10839 nor its *pilY1* transposon mutants were twitching (Supporting information, Fig. S1). Complementation *in trans* with the functional PAO1 *pilQ* gene conferred twitching to TBCF10839 to levels seen in reference strain PAO1, but did not restore twitching in the *pilY1* mutant (Fig. S1). Transposon insertions into the piliated and twitching-proficient PA14 strain (Rahme *et al.*, 1995) also led to the loss of twitching (data not shown). These findings confirm the report by Alm *et al*. (1996) that a functional PilY1 protein is necessary for twitching.

**Fig. 1:**
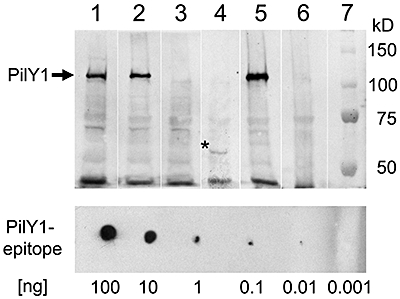
Upper panel: Immunoblot analysis of PilY1 in *P. aeruginosa* cell lysates. From left to right: PilY1-immunoreactive signals from planktonic cells of *P. aeruginosa* PAO1 (126.6 kDa), lane 1; TBCF10839 (126.3 kDa), lane 2; TBCF10839 *pilY1*::Tn*5* mutant 25C8, lane 3; TBCF10839 *pilY1*::Tn*5* mutant 10CB5, lane 4; TBCF10839 *pilY1*::Tn*5* mutant 25C8 complemented with pME6010::TB*pilY1*, lane 5; and TBCF10839 *pilW*::Tn*5* mutant 14D1, lane 6; grown until late exponential phase. Equivalent amount from cell lysates were subjected to 6% SDS-PAGE followed by immunodetection with rabbit polyclonal anti-PilY1 antibody (Eurogentec). The asterisk indicates the immunoreactive band of a truncated PilY1 protein in mutant 10CB5. Lower panel: Sensitivity of the rabbit polyclonal anti-PilY1 antibody against the PilY1-specific oligopeptide used for immunization.

As already the TBCF10839 host strain was phenotypically silent in twitching, we hypothesized that PilY1 should have further roles in *P. aeruginosa* beyond pilus-mediated motility. If this is the case, the PilY1 protein should be present in the non-piliated host. The *pilY1* gene of strain TBCF10839 was sequenced and highly sensitive and specific polyclonal peptide anti-PilY1 antibodies were raised against epitopes that are shared between the PilY1 orthologues of strains TBCF10839 and PAO1. PilY1-immunoreactive bands of predicted size (126 kDa) and similar intensity were detected on immunoblots of PAGE-separated cell lysates from strains PAO1 and TBCF10839 (Fig. 1) suggesting that PilY1 was present in similar amounts in the piliated and the non-piliated strain. The presence of PilY1 irrespective of the status of piliation provided the first evidence for roles of the PilY1 protein other than pilus biogenesis that should be associated with susceptibility to killing by PMNs as a surrogate function.

No PilY1-immunoreactive bands were seen in the cell lysates of the *pilW* mutant 14D1 (transposon insertion at position 497) and of the *pilY1* mutant 25C8 with the distal insertion of the transposon at position 2308 (Fig. 1). A faint band of about 59 kDa was recognized by the anti-PilY1 antibody in the *pilY1* mutant 10CB5 with the proximal insertion at position 1617 (Fig. 1, Fig. S2). This datum shows that the insertion of a transposon in *pilW* upstream of *pilY1* in the *fimU-pilVWXY1Y2E* operon had a strong polar effect and caused a knockout of PilY1. Mutagenesis within *pilY1* itself led either to a null mutant or to a leaky mutant with minute production of truncated protein. Complementation of the null mutant *in trans* led to overproduction of PilY1 (Fig. 1).

**Fig. 2:**
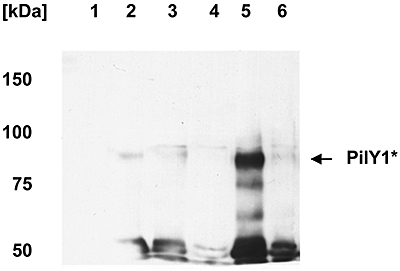
Immunoblot analysis of PilY1 in *P. aeruginosa* immunoprecipitates of the extracellular protein fraction that had been concentrated about a 100-fold by ultrafiltration. From left to right: PilY1-immunoreactive signals of the concentrated extracellular protein fraction of TBCF10839 *pilY1*::Tn*5* (25C8) (lanes 1, 2, 3) or of TBCF10839 (lanes 4, 5, 6) precipitated with beads (lanes 1, 4; negative control), rabbit polyclonal anti-PilY1(TB)-antibody (lanes 2, 5) or pre-immune serum from the same rabbit (lanes 3, 6; negative control). The anti-PilY1 antibodies solely detected the shorter PilY1* isoform, but no full-length PilY1 in the supernatant of TBCF10839 cultures (lane 5).

The strong PilY1-immunoreactive signals of PAO1 and TBCF10839 cell lysates shown in Fig. 1 were obtained from 2 ml of culture. The corresponding extracellular protein fractions that contain secreted proteins or sheared cellular appendages like pili were immunonegative for PilY1 on blots. To improve the sensitivity, supernatants of bacterial cultures were 100-fold concentrated for proteins larger than 50 kDa by ultrafiltration and then immunoprecipitated with anti-PilY1 antibody. A PilY1 signal of predicted size of the full-length protein was still not detectable on the blots. However, an immunoreactive band of about 88 kDa was visible in the immunoprecipitate from TBCF10839 (Fig. 2). The same band became apparent on blots of whole cell lysates of TBCF10839 after longer exposure with the ECL detection reagent, whereas blots derived from cell lysates of PA14 that lack the epitopes used for anti-PilY1-antibody generation, showed no signal (Fig. S2B). This datum demonstrates that the anti-PilY1 antibody recognized PilY1-specific epitopes in the 88 kDa protein. As the immunoreactive 88 kDa signal was seen neither in cell lysates of PA14 nor in the pre-immune control of the immunoprecipitations, this 88 kDa band most likely represents a shorter isoform PilY1* of strain TBCF10839. Only PilY1*, but not the full-length PilY1, was present in the extracellular protein fraction. As the PilY1*-immunoreactive signal became only visible by immunoprecipitation from concentrated supernatant, maximal 5%, but probably even less of total PilY1 resided in the extracellular fraction. No PilY1* signal was detected in the immunoblots of the TBCF10839 *pilY1* transposon mutant (see Fig. 1 and Fig. S2 for the cell lysate and Fig. 2 for the immunoprecipitate of concentrated supernatant).

### mRNA expression profiling

Next, evidence for the putative role(s) of PilY1 was sought by comparative mRNA expression profiling. RNA was extracted from strain TBCF10839 and its isogenic *pilY1* transposon mutant 25C8 grown in Luria–Bertani (LB) broth to late exponential phase, reverse-transcribed and hybridized onto *P. aeruginosa* PAO1 GeneChips. The mRNA expression profile of the transposon mutant in comparison with the wild-type strains showed the significant upregulation of 20 genes and significant downregulation of 56 genes (Supporting Information, Table S1). Genes *fimUpilVWX* upstream of *pilY1* were the most strongly upregulated genes indicative for a feedback regulation of expression of the whole operon that was destroyed by the insertion of the transposon. Of the 71 other genes that were classified as differentially regulated, only eight genes have yet been experimentally characterized. Within the group of moderately or strongly expressed genes that were differentially regulated, conserved hypotheticals and putative enzymes of unknown function had a share of more than 80%.

Hence, the higher mRNA transcript levels of PilY1 and of the minor prepilins FimU, PilV, PilW and PilX in the *pilY1* mutant (Table S1) were the only interpretable differences in the global expression profile between TBCF10839 and TBCF10839 *pilY1*::Tn*5*. This observation was verified by RT/PCR kinetics. mRNA transcript levels were measured in piliated PA14 and non-piliated TBCF10839 and their isogenic *pilY1* mutants (Fig. S3). PilW mRNA transcripts were about 40- and 20-fold and mutant PilY1 mRNA transcripts were about 10- and 50-fold more expressed in the transposon mutants than in their parental TBCF10839 and PA14 strains respectively.

### Secretion of secondary metabolites

*Pseudomonas aeruginosa* secretes numerous coloured secondary metabolites (Ramos, 2004). Both TBCF10839 and PA14 are strong producers of these compounds, but their isogenic *pilY1* mutants were colourless. Notably none of the known genes involved in the biosynthesis of secondary metabolites was differentially regulated in the TBCF10839 *pilY1*::Tn*5 (*25C8) transcriptome. Thus a process at the post-transcriptional level probably affected the secretion of these compounds in the *pilY1* mutants.

**Fig. 3:**
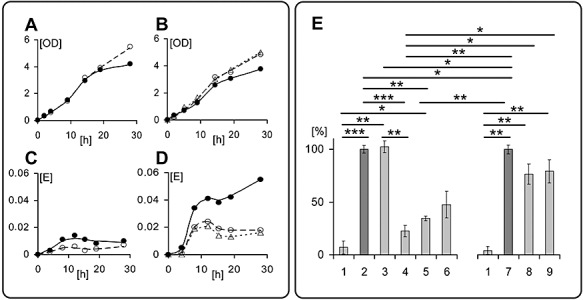
Time-course of growth and of pyocyanin secretion of TBCF10839, PA14 and their isogenic *pilY1* transposon mutants. Growth (A, B) of bacteria (37°C, 200 r.p.m., 50 ml medium) was monitored by the optical density of the bacterial culture at 578 nm. Pyocyanin (C–E) was determined spectrophotometrically at 695 nm in the spent supernatants. Panels A, C: closed circle, TBCF10839; open circle, TBCF10839 *pilY1*::Tn*5* (25C8); panels B, D: closed circle, PA14; open circle, PA14 *MAR2xT7 pilY1* mutant ID25263; open triangle, PA14 *MAR2xT7 pilY1* mutant ID25563. Panel E. Pyocyanin contents in supernatants of *P. aeruginosa* cultures grown to an OD of 2.5–3.0. The histogram shows the mean ± SD of five separate experiments. Values were normalized to the pyocyanin secretion of the TBCF10839 and PA14 wild-type strains respectively. 1, strain PAO1; 2, TBCF10839; 3, TBCF10839 complemented with pME6010 (vector control); 4, TBCF10839 *pilY1*::Tn*5* (25C8); 5, TBCF10839 *pilY1*::Tn*5* (25C8) complemented with pME6010; 6, TBCF10839 *pilY1*::Tn*5* (25C8) complemented with pME6010::TB*pilY1*; 7, PA14; 8, PA14 *MAR2xT7 pilY1* mutant ID25263; 9, PA14 *MAR2xT7 pilY1* mutant ID25563. Means of absolute values were examined for significant differences between two strains by two-tailed *t*-test and subsequent Bonferroni correction for multiple testing (*P < 0.05; **P < 0.01; ***P < 0.001).

To characterize this phenotype in more depth, the time-course of growth and of the secretion of the phenazine pyocyanin and of the siderophore pyoverdine were monitored for strains TBCF10839, PA14 and their *pilY1* mutants 25C8, 25263 and 25563 in King A and King B medium respectively. In both genetic backgrounds the growth curves uncovered two remarkable features of the mutant strains (Fig. 3). First, the *pilY1* mutants were growing to higher bacterial densities (panels A and B in Fig. 3), and second, they secreted less pyocyanin at all time points (panels C and D). Complementation of TBCF10839 *pilY1*::Tn*5* restored the secretion of pyocyanin (Fig. 3E). The production of pyoverdine was not affected by PilY1 deficiency (data not shown) which was consistent with the finding that the global production of siderophores on chromazurol iron indicator plates was similar for wild-type strains, *pilW and pilY1* mutants and their complemented revertants (Fig. S4B).

*Pseudomonas aeruginosa* possesses an extended network of *N*-acylhomoserine lactone (AHL) quorum-sensing (QS) systems that upregulate large, overlapping sets of genes including the pyocyanin biosynthesis gene cluster (Whiteley *et al*., 1999; Juhas *et al*., 2004; Schuster and Greenberg, 2006). Defective QS may improve fitness and growth in stationary phase (Heurlier *et al*., 2006). The stronger growth in stationary phase and the impaired secretion of pyocyanin of the PA14 and TBCF10839 *pilY1* mutants (see Fig. 3) thus mimics aspects of a QS-defective phenotype. Hence we checked AHL production in our strain panel. However, as shown in Fig. S5, all strains were similarly proficient in the production of short-chain and long-chain AHLs. In other words, inactivation of *pilY1* neither directly nor indirectly affected secretion of AHLs.

A major readout of AHL-mediated cell-to-cell communication is the stimulation of type II secretion virulence effectors such as elastases. Protease secretion can be screened on agar plates that contain casein as sole carbon source (Cowell *et al*., 2003). Figure S4A demonstrates that PA14, TBCF10839 wild type and its *pilW* and *pilY1* transposon mutants were proficient in casein degradation. In summary, there is no evidence that the AHL-dependent QS systems are perturbed in the *pilY1* mutants that could account for the impaired secretion of pyocyanin.

**Fig. 4:**
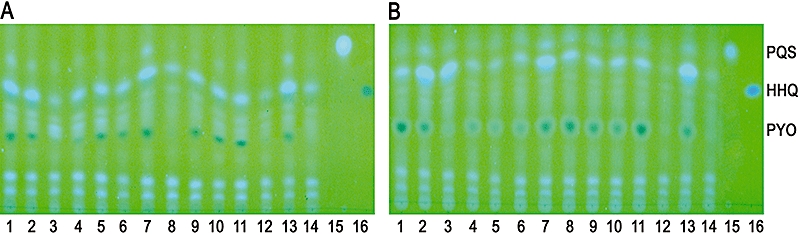
HAQ metabolite analysis. Thin-layer chromatogram of organic extracts of whole cultures grown to OD_578_ = 2.5 (A) or grown for 15 h (B) in LB broth of *P. aeruginosa* strains 1, TBCF10839; 2, PA14; 3, PAO1; 4, TBCF10839 *pilY1*::Tn*5* (10CB5); 5, TBCF10839 *pilY1*::Tn*5* (25C8); 6, TBCF10839 *pilW*::Tn*5*; 7, PA14; 8, TBCF10839 *pilY1*::Tn*5* (10CB5) complemented with pME6010::TB*pilY1*; 9, TBCF10839 *pilY1*::Tn*5* (25C8) complemented with pME6010::TB*pilY1*; 10, TBCF10839 *pilW*::Tn*5* complemented with pME6010::TB*pilW*; 11, TBCF10839 complemented with pME6010 (vector control); 12, TBCF10839 *pilY1*::Tn*5* (25C8) complemented with pME6010; 13, TBCF10839 *pilW*::Tn*5* complemented with pME6010. The chemically synthesized HAQs 3,4-dihydroxy-2-heptylquinoline (PQS) (lane 15) and 4-hydroxy-2-heptylquinoline (HHQ) (lane 16) were included as standards. All samples in A and B, respectively, were processed by the same operating procedure including identical quantities of volume at corresponding steps.

Besides phenazines and siderophores, the third major class of coloured secondary metabolites in *P. aeruginosa* that emit visible light by fluorescence are the 4-hydroxy-2-alkylquinolines (HAQs) which include antimicrobial N oxides and the cell-to-cell communication molecules 3,4-hydroxy-2-heptylquinoline [pseudomonas quinolone signal (PQS)] and 4-hydroxy-2-heptylquinoline (HHQ) (Pesci *et al*., 1999; Lépine *et al*., 2004; Xiao *et al*., 2006). Cultures of *pilY1* and *pilW* transposon mutants of TBCF10839 (Fig. 4B), but not cultures of *pilY1* mutants of PA14 (Fig. S6) grown for 15 h to late exponential phase contained lower amounts of HAQs than cultures of the wild-type strain. The more hydrophobic compounds including PQS were underrepresented. Supply of the *pilY1* or *pilW* gene in plasmid pME6010, but not plasmid alone, restored the composition of HAQs in TBCF10839 mutants to wild-type levels (Fig. 4).

**Fig. 5:**
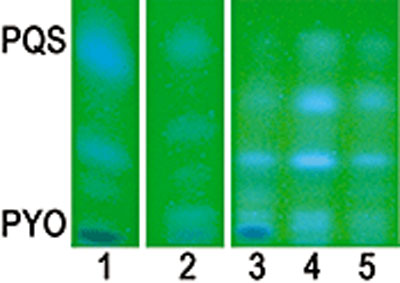
Thin-layer chromatogram of organic extracts of cell-free supernatants (lanes 1, 2) or of supernatants enriched for high molecular weight (> 50 kDa) compounds by ultrafiltration (lanes 4, 5) of cultures of strains TBCF10839 (lanes 1, 4) and TBCF10839 *pilY1*::Tn*5* (25C8) (lanes 2, 5) grown for 20 h. Lane 3, organic extract of a 20 h culture of TBCF10839. Assuming a 100% yield of extraction, the chromatogram represents the contents of HAQs and phenazines in 80 μl cell-free supernatant (lanes 1, 2), 100 μl cell culture (lane 3) and 1.7 ml concentrated supernatant (lanes 4, 5). The same pattern of compounds as in lane 4 was seen in the anti-PilY1 antibody immunoprecipitate that was analysed in parallel for PilY1-immunoreactive signals on blots (see Fig. 2, lane 5).

In wild-type cells HAQs were recovered in larger yields from the supernatant than from the cell pellet (Fig. S7). Extracts from the supernatant of TBCF10839 contained substantially larger amounts of PQS and pyocyanin (Fig. 5, lane 1) than those from the supernatant of isogenic *pilY1* mutants (Fig. 5, lane 2) indicating that the secretion of these hydrophobic compounds is decreased in the mutants (see also Fig. S7). *P. aeruginosa* packages PQS into membrane vesicles that serve to traffic this molecule within a population (Mashburn and Whiteley, 2005). Hence we hypothesized that like PQS all HAQs may aggregate in stable polydisperse structures in the extracellular aqueous medium. To resolve this issue, the fraction of more than 50 kDa molecular weight in the cell-free supernatant was concentrated by ultrafiltration. About 5% of the hydrophobic compounds in the spent supernatant were recovered in this fraction of multimeric aggregates. Pyocyanin was missing and unexpectedly PQS was also underrepresented in this fraction that could not pass a 50 kDa filter (Fig. 5, lanes 4 and 5). The same pattern of hydrophobic secondary metabolites as shown in lanes 4 and 5 was seen for thin-layer chromatography (TLC)-separated organic extracts of the retentate immunoprecipitated with anti-PilY1 antibody. This datum indicated that extracellular PilY1* was associated with HAQs.

In summary, the reduced secretion of pyocyanin was the major cause for colourless cultures of the PA14 and TBCF10839 *pilY1* mutants. The latter mutants also produced and secreted less HAQs.

### Colony morphology

**Fig. 6:**
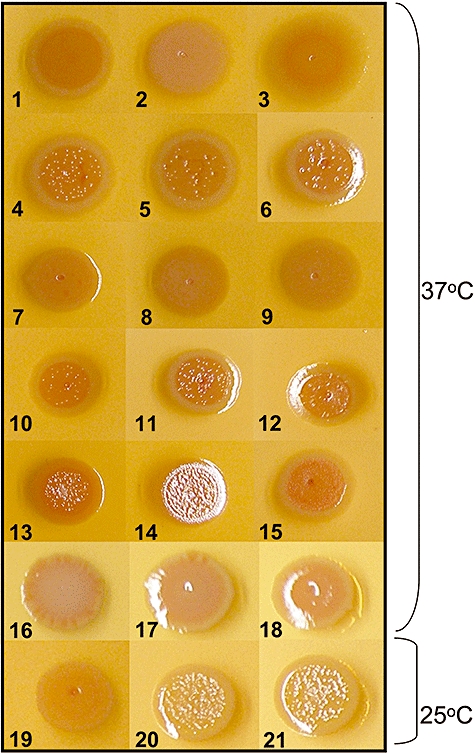
Colony morphology of *P. aeruginosa* strains grown for 24 h in ambient air on Congo red agar. Growth of PAO1, PA14 and isogenic TBCF10839 strains at 37°C (1–15): 1, TBCF10839; 2, PAO1; 3, PA14; 4, TBCF10839 *pilY1*::Tn*5* (25C8); 5, TBCF10839 *pilY1*::Tn*5* (10CB5); 6, TBCF10839 *pilW*::Tn*5*; 7, TBCF10839 *pilY1*::Tn*5* (25C8) complemented with pME6010::TB*pilY1*; 8, TBCF10839 *pilY1*::Tn*5* (10CB5) complemented with pME6010::TB*pilY1*; 9, TBCF10839 *pilW*::Tn*5* complemented with pME6010::TB*pilW*; 10, TBCF10839 *pilY1*::Tn*5* (25C8) complemented with pME6010 (vector control); 11, TBCF10839 *pilY1*::Tn*5* (10CB5) complemented with pME6010; 12, TBCF10839 *pilW*::Tn*5* complemented with pME6010; 13, TBCF10839 TB*pqsL*::Tn*5* (insertion of Tn*5* at position 541 of *pqsL*, autolysis induced by the overproduction of HAQs, D'Argenio *et al*., 2002); 14, TBCF10839 TB*phiCTXp40*::Tn*5* (insertion of Tn*5* at position 30 014 in ORF37 of phage ΦCTX (Nakayama *et al*., 1999), autolysis induced by bacteriophage); 15, TBCF10839 *pqsR*::Tn*5*[insertion of Tn*5* at position 639 of *pqsR* (*mvfR*), transcriptional regulator (Xiao *et al*., 2006) that induces the expression of the *pqsABCDE* operon, D'Argenio *et al*., 2002]. Growth of isogenic PA14 strains (16–21) at 37°C (upper panel) or at 25°C (lower panel). 16, 19, PA14; 17, 20, PA14 *MAR2xT7 pilY1* mutant ID25263; 18, 21, PA14 *MAR2xT7 pilY1* mutant ID25563.

Colonies of the TBCF10839 *pilW* and *pilY1* mutants showed at 42°C a wild-type morphotype and at 37°C autolysis in the centre (Fig. 6, no. 4–6). The autolytic morphotype was reverted to the non-autolytic colony morphology of the parental TBCF10839 strain (Fig. 6, no. 1) by complementation *in trans* with a *pilW* or *pilY1* containing plasmid (Fig. 6, no. 7–9), but not with plasmid alone (Fig. 6, no. 10–12). PA14 *pilY1* mutants showed a wild-type morphotype at 37°C (Fig. 6, no. 17 and 18) and autolysis at 25°C (Fig. 6, no. 20 and 21).

Autolysis has been described to be induced either by bacteriophage (see Fig. 6, no. 14) or by the uncontrolled overproduction of HAQs which can be mimicked by the inactivation of the *pqsL* (PA4190) gene (Fig. 6, no. 13) (D'Argenio *et al*., 2002). The morphotype of *pilY1* and *pilW* mutants did not resemble that of phage-induced lysis. Electron microscopic examination of colonies of the mutants (see below) did not observe any phage, whereas numerous phages were seen after transduction of strain PAO1 with phage F116 as positive control (Fig. S8). The *pilY1* and *pilW* mutant morphotype was also phenotypically different from that of a HAQ overproducer. The *pqsL* transposon mutant of TBCF10839 exhibited a patchy morphotype with lytic and non-lytic zones side-by-side throughout the colony (Fig. 6, no. 13). Colonies were glossy in the HAQ-overexpressing *pqsL* mutant. TBCF10839 *pilW* and *pilY1* mutants were poor HAQ producers (Fig. 4, Fig. S7) and correspondingly the colonies appeared matt (Fig. 6, no. 4–6). In summary, the colony morphology of *pilY1* and *pilW* mutants is different in phenotype from that of phage lysis or HAQ over-production and thus represents a novel, yet undescribed temperature-dependent autolytic morphotype.

### PilY1 is associated with small extracellular aggregates in electron microscopy

**Fig. 7:**
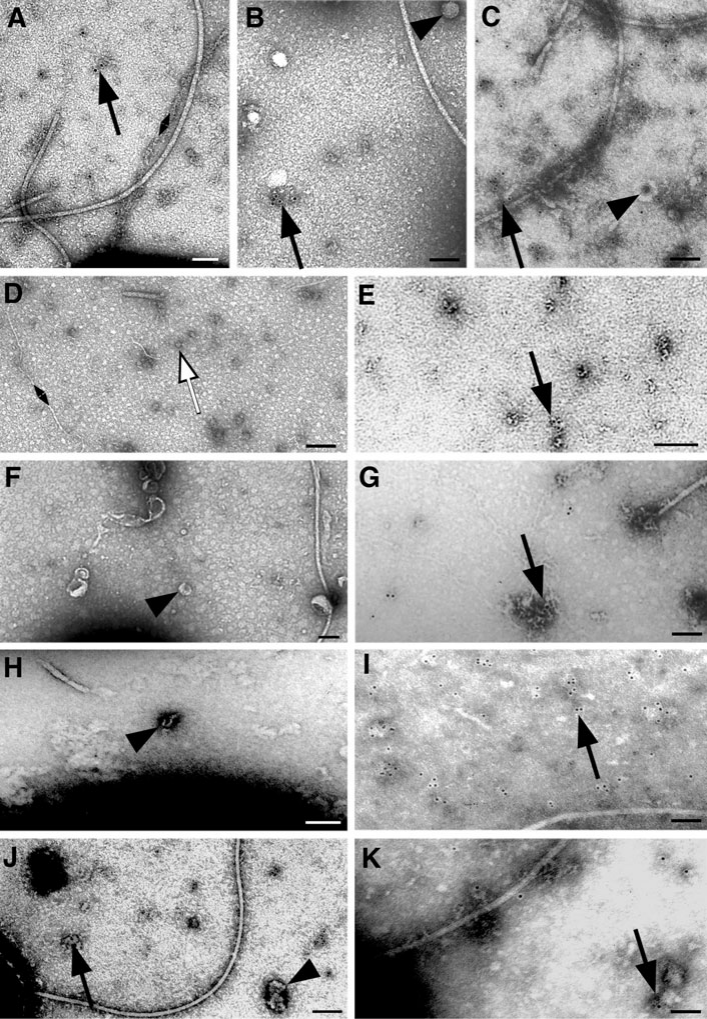
Electron microscopy of the extracellular milieu of *P. aeruginosa* strains PAO1 (A), TBCF10839 (B), TBCF10839 complemented with pME6010 (vector control) (C), TBCF10839 complemented with pUC20::PAO1*pilQ* (D, E), TBCF10839 *pilY1*::Tn*5* (25C8) (F), TBCF10839 *pilY1*::Tn*5* (25C8) complemented with pME6010::TB*pilY1* (G), TBCF10839 *pilY1*::Tn*5* (10CB5) (H), TBCF10839 *pilY1*::Tn*5* (10CB5) complemented with pME6010::TB*pilY1* (I), TBCF10839 *pilW*::Tn*5* (14D1) (J) and TBCF10839 *pilW*::Tn*5* (14D1) complemented with pME6010::TB*pilW* (K). Immunogold detection of PilY1 (A–C, E–K) was carried out using anti-PilY1 antibody from rabbits (Eurogentec) incubated with gold labelled anti-rabbit IgG (12 nm; Jackson Immunology Laboratory). Closed arrows point to small aggregates (‘nanoparticles’) in the presence of 12 nm gold- anti-PilY1 antibodies (A–C, E, G, I, K), open arrows point to small aggregates (‘nanoparticles’) in the absence of 12 nm gold- anti-PilY1 antibodies (D). Arrowheads point to round aggregates interpreted as collapsed vesicles (B, C, F, H, J). A rhombus points to fragmented pili (A, D). The bar indicates the size of 100 nm (original magnification 50 000).

To seek for extracellular structures that are present in PilY1-proficient, but absent in PilY1-deficient cells, cultures of wild-type strains, *pilW* or *pilY1* mutant and complemented strains were compared by anti-PilY1 immunogold electron microscopy (Fig. 7). Negative staining visualized various extracellular structures, namely cellular appendages (pili, flagella), vesicular structures and small aggregates, the latter of which was decorated with the anti-PilY1 antibody.

TBCF10839 bacteria were surrounded by 30–80 nm round aggregates (Fig. 7B). Isolated smaller irregularly formed extracellular products were decorated with the anti-PilY1 antibodies. PAO1 bacteria were surrounded by only very small amounts of vesicular structures. The PilY1 antibody exclusively decorated the small extracellular aggregates (Fig. 7A). Examination of 70 μm of pili did not reveal a single gold particle along or at the end of the pili in contrast to 130 particles that were counted in a 70 μm stretch of nearest neighbour aggregates. This datum demonstrates that in *P. aeruginosa* the PilY1 protein is not associated with any extracellular pilus structure (*P* = 10^−25^, Fisher's exact test), but with small aggregates. In piliated TBCF10839 complemented with the PAO1 *pilQ* gene the small aggregates were decorated with the PilY1 antibody gold particles as seen for the wild-type strain (Fig. 7E), but no immunoreactive signal was detected in association with the pili. Cultures with PilY1-deficient mutants exhibited collapsed vesicles 50–80 nm in diameter, but no small aggregates and no T4P structures (Fig. 7F and H). The PilY1 mutants complemented with a *pilY1* containing plasmid were surrounded by 30–50 nm thick round vesicles and collapsed structures and by very small aggregates that were decorated with the PilY1 antibody (Fig. 7G and I). The PilW mutant showed a few PilY1 antibody-positive aggregates (Fig. 7J), but on the other hand the complementation *in trans* with recombinant *pilW* plasmid led to fewer antibody-positive aggregates than the complementation of *pilY1* mutants with *pilY1* (compare Fig. 7K with Fig. 7G and I).

In summary, cells of all investigated *P. aeruginosa* strains were found to be surrounded by vesicular structures smaller than 100 nm in size. Pili were only detected in strain PAO1 and strain TBCF10839 complemented with the *pilQ* gene of strain PAO1. Cultures of PilY1-proficient cells, but not those of PilY1-deficient cells, contained very small extracellular aggregates (‘nanoparticles’) that were decorated by PilY1 antibody. As the immunoprecipitation experiments detected only the ∼88 kDa isoform PilY1*, but not full-length PilY1 in the concentrated extracellular protein fraction, the PilY1-immunoreactive signals in the electron microscopic examination should represent the localization of PilY1* in the extracellular medium.

The exact composition of the PilY1*-positive nanoparticles is not known, but according to our data they contain – besides probably further, yet uncharacterized components – PilY1* and the spectrum of TLC-separated hydrophobic secondary metabolites that were extracted from the immunoprecipitates of the > 50 kDa fraction of spent supernatants. According to the *R*_f_-values, this fraction contained HAQs including HHQ, but was free of pyocyanin and partially depleted of PQS.

### Impact of PilY1 on virulence in worms and mice

The *in vitro* analyses demonstrated that the *pilY1* mutants were growing better in stationary phase, were prone to autolysis and despite normal AHL production deficient in the secretion of pyocyanin. We wanted to test the impact of this phenotype on the virulence of the TBCF10839 strain *in vivo.*

**Fig. 8:**
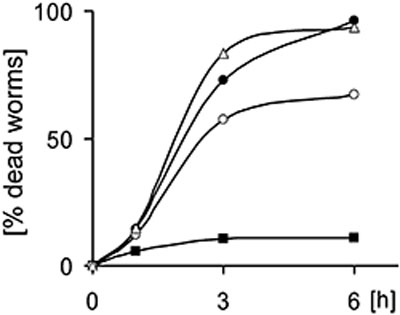
Kinetics of *C. elegans* fast-killing by *P. aeruginosa* TBCF10839 (closed circle), TBCF10839 *pilY1*::Tn*5* (25C8) (open circle) and TBCF10839 *pilY1*::Tn*5* complemented with pME6010::TB*pilY1* (open triangle)*. E. coli* DH5α served as negative control (closed square). The percentage of dead worms was scored by microscopic examination after 1, 3 and 6 h.

**Fig. 9:**
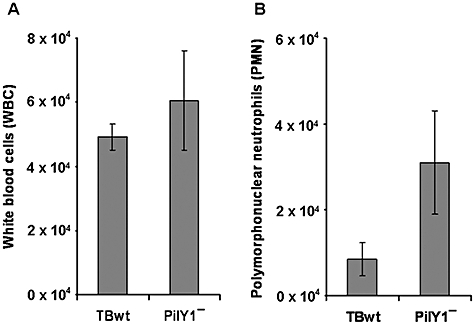
Inflammatory airway response of female C3H/HeN mice 4 days after intratracheal instillation of 8 × 10^5^ cfu of alginate-embedded *P. aeruginosa* TBCF10839 wild type (*n* = 6 mice) or TBCF10839 *pilY1*::Tn*5* mutant (*n* = 6 mice). Numbers of white blood cells and PMNs were scored in the BAL fluid from female C3H/HeN mice. Data are shown as mean ± SEM. The number of PMNs in BAL between the two groups of mice was significantly different (*P* = 0.013; *U*-test by Mann and Whitney).

**Fig. 10:**
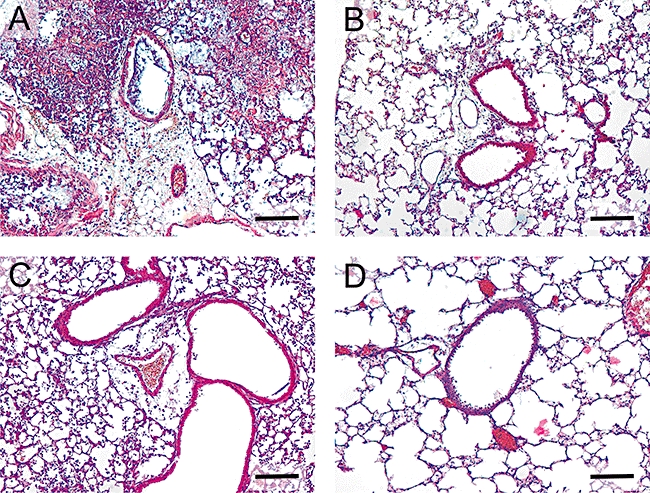
Pathohistological findings in C3H/HeN murine lungs 48 h after intratracheal infection with 7.5 × 10^6^ cfu of *P. aeruginosa* TBCF10839 (A), TBCF10839 *pilY1*::Tn*5* (25C8) (B), TBCF10839 *pilW*::Tn*5* (C), vehicle control (30 μl PBS) (D). Haematoxilin-eosin staining; original magnification ×100, scale bars 100 μm. TBCF10839 wild type (A) induces a profound purulent pneumonia with numerous foci of inflammation (infiltration of neutrophils and alveolar macrophages) up to tissue necrosis. In contrast the lungs of both isogenic mutants (B, C) show a moderate purulent alveolar pneumonia (neutrophils and alveolar macrophages in the alveoli but not within the bronchi). (D) Normal lung parenchyma after PBS application. The *in trans* complemented mutants were not investigated because the strains lose their episomal plasmids in murine lungs over time.

The ‘fast-killing’ of *Caenorhabditis elegans* has been ascribed to the pyocyanin-mediated production of reactive oxygen species (Mahajan-Miklos *et al*., 1999; Tan *et al*., 1999). In accordance with the literature reports, the pyocyanin-deficient TBCF10839 *pilY1* mutant was attenuated in the killing of the nematodes (Fig. 8). On the other hand, of more than 3500 screened TBCF10839 transposon mutants, the two *pilY1* mutants were most robust against killing by PMNs (see above). As PMNs represent the major recruited mammalian cellular host defence against *P. aeruginosa*, we hypothesized that the *pilY1* mutants should be endowed with higher fitness to colonize and persist in a mammalian host. To test this hypothesis, one *pilY1* and one *pilW* mutant and 23 other differentially tagged non-auxotrophic signature tagged mutagenesis (STM) TBCF10839 mutants were inoculated into murine airways by intratracheal instillation. Recovery of the surviving bacteria 48 h later demonstrated that the *pilW* and *pilY1* mutants were obtained in higher yields from the infected mouse lungs than from pools of the same mutants that had been cultured in parallel in LB broth *in vitro* (Fig. S9A). Competition experiments with two other sets of isogenic STM mutants showed again that the *pilW* and *pilY1* mutants had the highest fitness to persist and grow in murine airways (Fig. S9B and C). In parallel to the higher survival of the *pilY1* mutant in the murine habitat, the immigration of PMNs into the lungs was evoked in the mouse. A dose of 8 × 10^5^ alginate-embedded TBCF10839 bacteria or of its isogenic *pilY1* mutant 25C8 was instilled into murine airways. The bronchoalveolar lavage fluid collected 3 or 4 days after infection with the *pilY1* mutant contained higher number of white blood cells that could be attributed to the significant increase of PMNs (*P* < 0.05) (Fig. 9). The lack of PilY1 led to a stronger emigration of PMNs into the airways. The stronger attraction of PMNs to the infected site, however, was not associated with increased morbidity. When mice were challenged with the same doses of planktonic colony-forming units (cfu) of TBCF10839 wild type, TBCF10839 *pilW*::Tn*5* or TBCF10839 *pilY1*::Tn*5*, the signs of acute illness as indicated by the body condition score (Munder *et al*., 2005) and the intermittent drop of body weight and rectal temperature were lower upon exposure to the *pilW* or *pilY1* transposon mutant (Fig. S10). By day 2 the wild-type strain had caused a profound and the mutants a moderate pneumonia (see histopathology in Fig. 10). Although the transposon insertion into *pilY1* rendered the *P. aeruginosa* cell resistant to killing by the most important professional phagocyte, the global virulence of the strain was attenuated.

## Discussion

Signature tagged mutagenesis has been originally developed by David Holden and colleagues to identify new virulence genes of *Salmonella typhimurium* in animal infection models (Hensel *et al*., 1995), but during the last years the method has been adopted to genome wide scans of other bacteria in disease habitats and the environment (Shea *et al*., 2000; Mazurkiewicz *et al.*, 2006). The beauty of the technology is an *in vivo* selection process done by the habitat among a mixed population of mutants (Levesque, 2006). STM has the potential to discover novel and unexpected targets and pathways. Such a surprising finding was the starting point of this work. Screening of a STM library in a non-piliated *P. aeruginosa* strain uncovered that transposon inactivation of its *pilY1* gene rendered the bacterium more resistant against killing by PMNs than the wild type and any other of the more than 3500 tested mutants. PMN-mediated phagocytosis and killing is the most important mammalian host defence mechanism against *P. aeruginosa* (Döring *et al*., 1995) and hence we wanted to resolve the phenotypic commonalities and differences between PilY1-proficient and PilY1-deficient isogenic strains.

The *fimU-pilVWXY1Y2E* operon encodes five minor prepilins and two non-prepilin proteins, namely PilY1 and PilY2 (Belete *et al*., 2008). The prepilins are involved in export, maturation and presentation of the major fimbrial subunit, when these genes are expressed in the correct stoichiometric ratios (Alm and Mattick, 1995; 1996; 1997). The prepilins, but also PilY1 (see Fig. S1) are necessary to confer twitching motility. However, to date there are no reports that show the incorporation of the proteins in the T4P structure. This fits with our observation that the PilY1 protein was present in similar amounts in the piliated PAO1 and the non-piliated *pilQ*-defective TBCF10839 strains (Fig. 1).

The *pilY1* gene has been annotated to encode the tip-associated adhesin (Pseudomonas database http://www.pseudomonas.com; Stover *et al*., 2000) like its homologue in *N. gonorrhoeae* PilC (Rudel *et al*., 1995). Figure S11 shows a dendrogram of the closest currently known homologues of PilY1 based on CLUSTALX protein sequence alignments. The neisserial PilC and the *P. aeruginosa* PilY1 proteins form separate branches. The substantial sequence diversity may result in different functions, and indeed no PilY1-immunoreactive signals were associated with the extracellular parts of PAO1 pili in electron microscopy (Fig. 7). Hence, the claim in the database is not supported that PilY1 in *P. aeruginosa* is a tip-associated adhesin.

*Neisseria gonorrhoeae* PilC1 and PilC2 are multifunctional proteins in correspondence with their dual localization in cellular appendages and the bacterial outer membrane (Rahman *et al*., 1997), i.e. they promote adhesion, piliation and transformation competence that are executed by different modules (Morand *et al*., 2001). This study demonstrates that *P. aeruginosa* PilY1 is also a multifunctional protein. First, as shown in this and previous work (Alm *et al*., 1996), PilY1 is indeed essential for piliation. Second, and this is new, PilY1 of *P. aeruginosa* is involved in the release of secondary metabolites into the extracellular space and is necessary to prevent autolysis in a bacterial colony. The latter two functions of PilY1 were observed in both piliated and non-piliated strains and hence are independent of pilus assembly.

The anti-PilY1 antibody recognized, besides the full-length PilY1, a shorter isoform PilY1*. The specificity of the PilY1* signal was corroborated by the immunonegative blots of isogenic PilY1 knockouts and of strain PA14 whose PilY1 protein does not contain the epitopes against which the antibody was raised (Fig. 1 and Fig. S2). PilY1* was the only PilY1 isoform in immunoprecipitates of the extracellular protein fraction of TBCF10839. Hence PilY1* should be the PilY1 isoform that was visualized in the extracellular nanoparticles by immunogold electron microscopy. The particles were absent in *pilY1* knockout mutants which points to a functional association between PilY1* and the make-up of these structures. The PilY1 immunoprecipitates of the concentrated > 50 000 molecular weight extracellular fraction contained numerous hydrophobic compounds including HAQs. As these compounds have a molecular weight below 1000, they coprecipitated as polydisperse aggregates and thus most likely reflect the secondary metabolite components of the PilY1*-positive nanoparticles. Pyocyanin was completely and PQS was largely lacking from the particles. The signal molecule PQS is known to be extruded by vesicles (Mashburn and Whiteley, 2005), but the other major HAQs are translocated by other means across the bacterial membranes. Hence we conclude that PilY1 and/or PilY1* is involved in the discharge of these secondary metabolites. The transport was accomplished by both the piliated PAO1 and the non-piliated *pilQ*-defective TBCF10839 strains, suggesting that this function does not require a properly elongating T4P.

PilY1 is transported to the periplasm in a Sec-dependent manner (Goldberg *et al*., 2008) in accordance with their presence in the membrane fraction of a cell lysate. To be released into the extracellular medium, a small portion of the PilY1 pool may be carried by the T4P all the way to the external medium or may be expelled by the brief elongation of the type II pseudopilus as it is the case of type II-dependent exoproteins. As the nanoparticles are present in the extracellular medium of piliated and non-piliated *P. aeruginosa* (Fig. 7), the latter rather than the former mechanism should account for the release of PilY1* and secondary metabolites into the extracellular space. Although we have yet no experimental evidence for this, it is tempting to assume that prior to or during this process PilY1 is cleaved to the extracellular isoform PilY1*.

Transposon insertions in TBCF10839 *pilW* and *pilY1* caused similar phenotypes. The insertions may have affected the expression of the individual genes within the *fimU-pilVWXY1Y2E* operon to differential extent, but with respect to PilY1 protein the *pilW* and *pilY1* mutants caused the same phenotype as judged by immunoblot analysis (Fig. 1). Both mutants were PilY1 knockouts. Likewise, macroscopic phenotypes such as the autolytic morphotype (Fig. 6), pyocyanin and HAQ deficiencies (Fig. 4) and persistence in (Fig. S9) and inflammation of murine airways (Fig. 10) were indistinguishable between the examined *pilW* and *pilY1* mutants. Subtle differences were visualized in the electron micrographs (Fig. 7). Some extracellular PilY1* was seen in the *pilW* mutant at far below wild-type levels (Fig. 7J).

PA14 *pilY1* and TBCF10839 *pilY1* transposon mutants were growing to higher cell densities than their parental strains. PA14 mutants appeared colourless, but their HAQ and pyocyanin deficiencies at 37°C were less pronounced (Fig. S6) than those of the TBCF10839 mutants (Fig. 4). Interestingly, the PA14 mutants showed autolysis at 25°C, but not 37°C, whereas the TBCF10839 mutants showed autolysis at 37°C, but not at 42°C. This temperature dependence points to a delicate interplay between growth and secretion of secondary metabolites that is modulated by the genetic background with PilY1 being a major modifier to prevent cell lysis. According to our data PilY1 seems to be involved in the control of both cell density (Fig. 3) and secretion (Figs 3–5, S6, S7). Cell density is monitored by the QS network (Juhas *et al*., 2004). AHL levels were normal in the *pilY1* mutants (Fig. S5) and thus signalling via the *las* and *rhl* networks probably remained unaffected in PilY1-deficient cells. The levels of PQS and pyocyanin, however, were lower in the mutants so that the PQS and PYO regulons operating in the late exponential and early stationary phase (Dietrich *et al*., 2006) could principally be affected in the *pilY1* mutants. The transcriptome, however, provided no evidence for any direct or indirect perturbation of the QS regulons in the TBCF10839 *pilY1* transposon mutant (Table S1). Hence, the inactivation of *pilY1* exerts its effects at the post-transcriptional level. The experimental data point to direct roles of PilY1 at the interface between cell and environment. Besides the assistance in secretion and pilus biogenesis, the annotated role of PilY1 as an adhesin may be materialized by the control of growth during stationary phase. The cell-associated PilY1 and the extracellular nanoparticle-associated PilY1* could scan cell density by scoring cell–cell, cell–matrix or matrix–matrix contacts mediated by PilY1–PilY1, PilY1–PilY1* or PilY1*–PilY1* interactions respectively. These contacts between PilY1/PilY1* molecules could confine the density of the stationary bacterial population. This scenario is similar to the dual roles of numerous proteins involved in territorial stability in multicellular organisms that either exist in the membrane or are processed by proteolytic cleavage and then released to become part of the extracellular matrix. The inactivation of PilY1 will lead to the observed stronger growth in stationary phase and finally autolysis at high cell densities, but on the other hand it may facilitate persistence in hostile habitats like the murine lung or lysosomal compartments of a PMN.

In the latter case, the intracellular retention of HAQs like PQS (Häussler and Becker, 2008) may help to counterbalance the oxidative stress exerted by the neutrophil. When the phagocytosed mutant cells are delivered to the phagolysosome, the lysosomal enzymes will lyse the bacterial cells. HAQs are released into the lysosomal compartment. The noxious bacterial compounds will damage the PMN and thereby impede bacterial cell killing. Consequently PilY1-defective *P. aeruginosa* cells will survive better during a standardized PMN phagocytosis assay *in vitro.* On the other hand, if the toxic effect is exerted by viable bacteria via the release of pyocyanin, the eukaryotic host will be less harmed by an attack of pyocyanin-deficient PilY1 mutants. An example is the *C. elegans* fast-killing infection model where the secretion of pyocyanin by viable bacteria is instrumental for virulence (Tan *et al*., 1999) (see Fig. 8).

In summary, *P. aeruginosa* PilY1 has a role in the control of cell density in the bacterial population and assists in pilus biogenesis, twitching motility and the release of secondary metabolites into the extracellular space. PilY1 shows substantial amino acid sequence diversity particularly in the N-terminal regions (Fig. S11). In conjunction with divergent genetic backgrounds, this may modulate phenotype and fitness traits of clones in the *P. aeruginosa* population (Wiehlmann *et al*., 2007a).

Inactivation of *pilY1* in the CF isolate TBCF10839 rendered the bacterial cells more resistant to killing by PMNs and improved their persistence in murine airways. Future studies should reveal whether *pilY1* or the other genes in the operon are targets for mutagenesis in natural infections with *P. aeruginosa* and may confer an advantage or disadvantage in fitness to colonize and to persist in animate habitats and to breach epithelial barriers.

## Experimental procedures

### Strains, plasmids and growth conditions

Plasmids, strains and culture conditions are listed in Table S2. The *P. aeruginosa* PA14 mutants ID 25563 and 25263 with transposon insertions at different positions of the *pilY1* gene were recovered from the PA14 transposon library (Liberati *et al*., 2006). The *P. aeruginosa* TBCF10839 STM transposon library was constructed with the plasposon pTnModOGm (Dennis and Zylstra, 1998) carrying variable signature tags as described previously (Wiehlmann *et al*., 2007b; Rakhimova *et al*., 2008). *P. aeruginosa* or *Escherichia coli* strains were routinely grown overnight as shaken cultures (230 r.p.m.) in LB broth medium at 37°C. Five millilitre LB broth was inoculated with a toothpick of frozen bacterial stock solution and incubated for 16–48 h. Recombinant *E. coli* DH5α strains transfected with pME6010 (Heeb *et al*., 2000) were growing in the presence of 50 μg ml^−1^ tetracycline and recombinant *P. aeruginosa* were cultured in the presence of 200 μg ml^−1^ tetracycline. *P. aeruginosa* PAO1 was transduced with phage F116 as follows (Maillard *et al*., 1995; Byrne and Kropinski, 2005): Phage F116 and 200 μl of an overnight culture of *P. aeruginosa* PAO1 were inoculated into 20 ml LB broth in a 100 ml flask and incubated for 2 h at 37°C with shaking (200 r.p.m.). After the addition of 40 μg mitomycin, the incubation was continued for another 30 min. The cells were pelleted by centrifugation at 4°C. The pellet was dissolved in 20 ml fresh LB broth and incubated for a further 2 h (37°C, 200 r.p.m.). Cells were pelleted by centrifugation at 4°C, and the supernatant was filtrated through a sterile 0.2 μm filter to harvest the phages. For electron microscopy, 10 μl each of this phage stock solution was dropped on single PAO1 colonies that had been grown overnight on agar plates.

### Colony morphology

Colony morphology was visualized on Congo red (40 μg ml^−1^) agar plates (Freeman *et al*., 1989). Three microlitre *P. aeruginosa* cultures from late stationary phase were inoculated onto the plate and incubated at 25°C or 37°C for 24–48 h. Autolysis was documented after 24–48 h.

### Twitching motility assay

Twitching motility was assayed according to the protocol by Alm *et al*. (1996). Briefly, bacteria from liquid culture were subsurfacely inoculated on LB agar allowing bacterial locomotion at 37°C. After 24 h the twitching zone on the Petri dish was visualized by Coomassie staining.

### DNA preparation

Genomic DNA from *P. aeruginosa* was isolated according to a protocol optimized for Gram-negative bacteria (Chen and Kuo, 1993).

### Complementation of *pilW* and *pilY1* transposon mutants

DNA fragments containing the *pilW* (PA4552) or *pilY1* (PA4554) genes were amplified from the genomic DNA of *P. aeruginosa* TBCF10839 and ligated into the BglII- and EcoRI-site of the shuttle vector pME6010 (Heeb *et al*., 2000). Introduction of the plasmid construct into strains TBCF10839 PA4554::Tn*5* or TBCF10839 PA4552::Tn*5* was carried out by electroporation. Revertants were selected on LB agar containing 200 μg ml^−1^ tetracycline. Sequencing of the PCR products was performed by Qiagen (Hilden).

### Fractionation of extracellular, intracellular and surface proteins

Whole cell lysates of *P. aeruginosa* were prepared by resuspending the bacterial pellet of a 2 ml culture in SDS-PAGE loading buffer followed by denaturation at 97°C for 4 min.

The extracellular protein fraction was prepared by two different procedures. Either the bacterial cell free supernatant of a 2 ml culture was precipitated by 70% EtOH or 10% (NH_4_)_2_SO_4_ at −21°C or 4°C respectively (Nilsson *et al*., 2000; Riedel *et al*., 2006). The protein pellets were collected by centrifugation (20 000 *g*, 30 min) and washed once with ice-cold ethanol. Air-dried pellets were resuspended in SDS-PAGE loading buffer for further immunoblot analysis. Alternatively, *P. aeruginosa* strains were cultured in 0.4 l tryptone/NaCl broth at 37°C with 150 r.p.m. until early stationary phase (OD_578_∼ 1.5–1.7). The bacteria were repetitively pelleted (first, 6000 *g*, 10 min, 4°C; then three times 8000 *g*, 15 min, 4°C). The supernatant was collected and then its macromolecular contents were concentrated by about 100-fold by centrifugation (2000 *g*) through a membrane filter (Vivaspin 20, cut-off 50 kDa, Sartorius). Aliquots of the retentate were analysed for its contents of HAQ by TLC (see below) or of PilY1 by immunoprecipitation. Ten microlitre affinity-purified anti-PilY1 rabbit polyclonal antiserum, 25 μl protein A agarose (Santa Cruz Biotechnologies) and 10 μl protein G agarose (Santa Cruz Biotechnologies) were sequentially added to 500 μl retentate and incubated overnight at 4°C. In parallel, further 500 μl aliquots of retentate were processed with either the pre-immune serum of the anti-PilY1 antiserum (1:1300 dilution) or beads only (negative controls). After centrifugation (5 min, 1000 *g*, 4°C) the beads were washed with PBS. After addition of 18 μl 3× Laemmli buffer (7% SDS (w/v)), the bead solution was incubated for 5 min at 95°C and then applied to 6% SDS-PAGE. The gel-separated immunoprecipitated proteins were then probed for anti-PilY1-immunoreactive signals by immunoblot as described below.

### PilY1 immunoblot analysis

Equal amounts of proteins from whole cell lysates or extracellular protein supernatant precipitates were separated on 6% SDS-PAGE and blotted onto nitrocellulose membrane. Detection of PilY1 with an affinity-purified anti-PilY1 rabbit polyclonal antiserum (1:1000) that had been raised against linear epitopes of PilY1 in PAO1 and in TB (pos. 338–352 CLPDGKSYSSQTPYRD) (Eurogentec) was carried out using the ECL Western Blot detection kit according to the manufacturer's protocol (Amersham). Pre-immune serum (1:500) did not show any immunoreactive signal against 100 ng peptide epitope or less. The sensitivity threshold of the used antiserum to the pilY1 peptide was 1 ng (Fig. 1).

### Immunogold electron microscopy

Bacteria were grown on LB agar plates. After 7.5 h microcolonies were collected on grids with carbon-coated formavar films. For negative staining the grids were floated on water and then on 1% aqueous uranyl acetate (Merck, Darmstadt, Germany) for 1 min respectively. For immunostaining the blocking reaction was performed in 3% (w/v) bovine serum albumin in phosphate buffered saline (PBS). The incubation on ice started with an affinity-purified rabbit antibody against PilY1-peptide diluted 1:30 in 3% bovine serum albumin in PBS for 40 min. After washing three times with PBS the grids were incubated with 12 nm colloidal gold-affinipure goat anti-rabbit IgG (Jackson Immunoresearch, West Grove, PA, USA) for 40 min followed by the negative staining as described before. The grids were examined in a Philips EM 301 electron microscope. The electron micrographs were selected, digitized and processed using Adobe photoshop 6.0.

### mRNA expression analyses

Whole genome mRNA expression profiling: RNA extraction, preparation of samples, hybridization of the *P. aeruginosa* PAO1 microarray and evaluation of data were performed according to the recommendations of the manufacturer and in-house protocols (von Götz *et al*., 2004; Salunkhe *et al*., 2005a,b). For semiquatitative RT/PCR assays, RNA was extracted from 6 × 10^8^ cfu of bacteria grown to late exponential phase (OD_578_∼ 2.5) using the Qiagen RNeasy Kit according to the manufacturer's instructions. DNA contaminations were removed by treatment with RNAse-free DNAse. cDNA was synthesized from 500 ng RNA by reverse transcription with random primers (AffinityScript QPCR cDNA Synthesis Kit, Stratagene). For PCR kinetics, a 1/400 aliquot of the resulting cDNA was amplified in a 50 μl reaction mixture (5 μl 10× reaction buffer (Eurogentec), 3.3 μl 25 mM MgCl_2_, 1 μl DMSO, 10 μl primer solution (5 μM each), 3 μl dNTPs (2 mM each). 1 U Goldstar-DNA-Polymerase (Eurogentec)). Primers are listed in Table S2. Aliquots were withdrawn every two cycles, separated by electrophoresis and stained with ethidium bromide. The relative amounts *N*_*i*_ and *N*_*j*_ of the template cDNA sequences *i* and *j* in the reaction mixture were determined from the titration for the first reaction cycle *n* when the PCR products became visible by ethidium bromide fluorescence during late exponential phase of PCR according to the equation


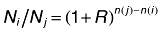
(1)

whereby the efficiency *R* of the used thermocycler during the exponential phase of PCR was determined to be *R* = 0.78 ± 0.02 for PCR products of 100–800 bp in length within the interval of reaction cycles 10 < *n* < 35 (Bremer *et al*., 1992).

### Pyocyanin and pyoverdine secretion

*Pseudomonas aeruginosa* strains were grown with shaking at 37°C in King A or King B medium (King *et al*., 1954) that preferentially stimulate the production of either pyocyanin (King A) or pyoverdine (King B). Cells were sedimented by centrifugation at 7000 *g* for 10 min. The absorption of the supernatant was measured spectrophotometrically at 695 nm (pyocyanin) or at 380 nm (pyoverdine).

### Plate assays of siderophore and protease secretion

Siderophore secretion was assessed by an orange halo around colonies grown for 24 h at 37°C on chromazurol plates as described by Schwyn and Neilands (1987). Secretion of casein-degrading proteases was examined by growing the analysed *P. aeruginosa* strains on M9 agar plates supplemented with 0.8% (w/v) casein (Cowell *et al*., 2003).

### Detection and characterization of AHLs

Bacteria were grown in LB broth to late exponential phase (OD_578_∼ 2.5). The AHL molecules were extracted with dichlormethane from spent culture supernatants of the strains. The extract was dried and redissolved in 0.2 ml ethyl acetate. An aliquot of 10 μl was separated by TLC and visualized by overlaying the TLC plates with soft agar seeded with the *luxAB* based AHL biosensor *E. coli* MT102 (pSB403) (detects long-chain and short-chain AHLs; Winson *et al.*, 1998), biosensor *Chromobacterium violaceum* CV026 (high sensitivity for short-chain AHLs; McClean *et al.*, 1997) or biosensor *Pseudomonas putida* F117 pKR-C12 (high sensitivity for long-chain AHLs; Steidle *et al.*, 2001). Bioluminescent spots indicating AHL were detected by exposure of a X-ray film. On the basis of their mobilities (*R*_f_-values) and by including appropriate reference compounds a tentative identification of AHLs present in the culture extracts was possible.

### HAQ detection

*Pseudomonas aeruginosa* strains were grown in a 50 ml flask in 10 ml LB broth with constant shaking at 37°C for 15 h or up to an optical density of OD_578_ = 2.5. One millilitre of the culture was extracted with 2 ml dichlormethane by vigorous shaking and the liquid phases were separated by centrifugation at 5000 *g* for 10 min. One millilitre of the organic phase containing HAQs and pyocyanin as major components was dried by evaporation. The pellet was resuspended in 50 μl methanol. Eight microlitres thereof was separated on Silica Gel 60 F254 TLC plates (that had been pre-soaked for 30 min in 5% (w/v) aqueous KH_2_PO_4_ solution and then dried for 60 min at 80–90°C prior to use) with 5% methanol/95% dichlormethane as the mobile phase. Fluorescent spots were visualized under UV light and photographed. PQS was synthesized from HHQ by the procedure described by Pesci *et al*. (1999) and used as standard.

### Phagocytosis assay in granulocytes

Bacteria were grown in LB medium at 37°C for 16 h. Granulocytes were prepared from the blood of healthy donors. Approximately 10 ml of fresh blood (with 100 I.U. heparin) was used for each experiment. The blood was mixed with 5 ml 10% hydroxyethyl starch and most of the erythrocytes were separated by sedimentation (45 min, room temperature). The granulocytes were then separated by centrifugation (3000 *g*, 15 min) using a lymphocyte separation medium (Lymphoprep, Axis-Shield, Oslo, Norway). The cell pellet was dissolved in 0.2 ml distilled water for 10 s to lyse residual erythrocytes. After mixing with 2 ml 1.1× PBS the cells were pelleted again by centrifugation. The sedimented granulocytes were resuspended in 2 ml RPMI1640 medium and stored on ice. Granulocytes were counted in a Neubauer chamber. 0.2 ml AB- blood serum and a 10-fold excess of bacteria (determined by photometry 0.6 OD_578_ = 10^9^ cfu ml^−1^) were added to the granulocyte suspension. This assay was incubated for 120 min (37°C, 200 r.p.m.). After incubation, the granulocytes with the internalized bacteria were separated from extracellular bacteria by centrifugation at 800 *g* for 10 min, resuspension in 0.3 ml RPMI1640, filtration (nitrocellulose, pore size 2 μm, Sartorius), and washing with PBS. The filters with the adhered granulocytes were transferred into distilled water for lysis and mixed vigorously for 5 min. The bacteria were transferred to new tubes and centrifuged (4000 *g*, 10 min). The supernatant was discarded and the pelleted bacteria were plated on LB agar. Survival of different *P. aeruginosa* strains was determined by cfu counts.

### Screening of pools of STM mutants for survival in the PMN phagocytosis assay or in the acute airway infection model in mice

Transposon mutants with different signature tags were separately grown in LB (37°C) overnight and pooled directly before the experiment.

In case of phagocytosis assays, aliquots of a 10-fold excess of bacteria to granulocytes were tested in parallel in phagocytosis assays in the absence (control I) or presence of PMNs (experiment type I). After 2 h of incubation, extracellular bacteria were removed by filtration through 5 μm nitrocellulose filters. Intracellular bacteria were released from the retained PMNs on the filter by the addition of 3 ml distilled water and vortexing for 5 min. The bacterial suspension was pelleted (4000 *g*, 10 min), resuspended in 0.1 ml PBS and plated on LB agar. Control I bacterial suspension was plated in parallel. Bacteria were collected after overnight incubation at 37°C.

In the mouse infection experiments, 100 μl of bacterial suspension was cultivated on LB agar or liquid for 48 h at 37°C (control II). Thirty microlitres (7.5 × 10^6^ cfu) was used for the intratracheal mice infection (experiment type II). Inoculation of bacteria and maintenance of mice are described in *Murine infection experiments*. After 48 h of infection, mice were sacrificed and the lungs were homogenized. Bacteria from the homogenised lungs were recovered on LB and LB agar at 37°C overnight. In parallel, bacteria from the control plates were collected and incubated on LB and LB agar at 37°C overnight in the same incubator.

Genomic DNA was prepared from both control and experiment and a PCR was performed to amplify the signature tags [primers P1 and P2 (Table S2; 35 cycles; 20 s, 58°C; 20 s, 72°C; 30 s, 94°C; 10 s ramp between each step)]. The PCR products were digested for 16 h with HindIII and the specific 40 bp sequence tags were separated from the common flanking 20 bp sequences by PAGE [10% gel (19+1 acrylamide/bis-acrylamide) in TBE buffer]. The 40 bp sequence tags were cut out from the gel and purified (QIAGEN). The 40 bp sequences were labelled with DIG-ddUTP using a terminal transferase (Roche) and hybridized onto dot blots. The dot blots were prepared as follows: 80 μl of the PCR products amplified from each single mutant was mixed with 40 μl of 3 M NaOH and 280 μl of TE buffer and denatured at 65°C for 30 min. After transfer to ice, 400 μl of 2 M CH_3_COONH_4_ was added and after short incubation, an aliquot of 95 μl of the DNA solution was applied to a Minifold-DOT-vacuum-blot device (Schleicher and Schuell) to immobilize the DNA on a Hybond N^+^ membrane. Dried and cross-linked membranes were pre-hybridized with 10 ml hybridization buffer [0.5 M NaH_2_PO_4_ × 2H_2_O, 7% SDS, 1 mM EDTA, 0.5% blocking reagent (Roche), pH 7.2] for 2 h at 58°C and afterwards hybridized with 40 ml of hybridization buffer containing denatured DIG-labelled probes for 16–24 h at 58°C in a hybridization oven. Non-specifically bound probe solution was removed by several washing steps and hybridization signals were detected by washing the membrane with anti-DIG-alkaline phosphatase (Roche) and CDP Star. Chemoluminescence signals were detected on X-ray films and quantified by PCBAS, version 2.09f (raytest Isotopenmeßgeräte GmbH). Signal intensity of each dot was compared with that of the corresponding signal of the probe prepared from pooled bacteria grown in parallel on LB agar without *in vivo* selection. Hybridization signals out of the 95% confidence interval of the mean were interpreted to be significantly different from the average signal.

### Sequencing of transposon flanking genes

Transposon-flanking sequences were amplified by the Y-linker method (Kwon and Ricke, 2000). Oligonucleotide sequences (Y-linker, Tn*5*MOD) are listed in Table S2. The Y-linker was prepared by the annealing of two oligonucleotides, Y1 and Y2. Genomic DNA of the mutants in aliquots of 1 μg was cut with 5 U NleIII or SphI (New England Biolabs) for 3 h and ligated to the phosphorylated and annealed Y-linker with T4-DNA ligase at 25°C for 2 h. The resulting product was used as a template in a PCR with the Y- and pTnMOD-specific primers. The resulting PCR products were purified by agarose gel electrophoresis and extracted using a Qiagen Gel extraction Kit. Sequencing was done by QIAGEN using the pTnMOD-specific primer. The raw sequences were analysed by blastn search against the sequences of the predicted genes as well as the complete genome sequence of *P. aeruginosa* PAO1 (Stover *et al*., 2000) or PA14 (Lee *et al*., 2006).

### Fast-killing of *C. elegans* (*Tan* et al*., 1999*)

*Caenorhabditis elegans* Bristol N_2_ (wild type), provided by the *Caenorhabditis* Genetics Centre (University of Minnesota, St Paul's, MN, USA), was maintained on nematode growth medium with *E. coli* OP50 as a food source. A total of 1.5 × 10^3^*P. aeruginosa* cells in 100 μl were plated (1% (w/v) peptone, 1% (w/v) NaCl, 1% (w/v) glucose, 0.15 M sorbitol, 1.7 (w/v)% agar) and incubated overnight at 37°C. Fifty synchronized *C. elegans* nematodes (stage N4) were added onto the plates and the number of dead worms was counted after 1, 3 and 6 h. Plates inoculated with *E. coli* OP50 served as negative controls.

### Murine infection experiments

#### Acute infection with planktonic P. aeruginosa

Bacteria were grown in LB broth overnight at 37°C (230 r.p.m.) to stationary phase. The bacteria were pelleted by centrifugation (5000 *g*, 10 min), washed twice with sterile PBS and the optical density of the bacterial suspension was adjusted by spectrophotometry at 578 nm. The intended number of cfu was extrapolated from a standard growth curve, and appropriate dilutions with sterile PBS were made to prepare the inoculum for the mice. To verify the correct dilution, an aliquot was serially diluted on LB agar plates. Ten- to twelve-week-old female mice of the inbred strain C3H/HeN (Charles River, Sulzfeld, Germany) were inoculated with 30 μl of the bacterial suspension via view controlled intratracheal instillation. This non-invasive application technique via catheter allows controlled delivery of the bacteria to the lungs (Munder *et al*., 2002). During the experiments mice were maintained in microisolator cages with filter top lids at 21 ± 2°C, 50 ± 5% humidity and 12 h light-dark-cycle. They were supplied with autoclaved, acidulated water and fed *ad libitum* with autoclaved standard diet. Prior to the start of the experiments animals were acclimatized for at least seven days. In case of 14-day infection experiments, the weight and rectal temperature of the mice were measured daily and their body condition was determined using a self-developed score (Munder *et al*., 2005). Murine behaviour was scored for the parameters vocalization, piloerection, attitude, locomotion, breathing, curiosity, nasal secretion, grooming and dehydration.

All animal procedures were approved by the local District Governments and carried out according to the guidelines of the German law for the protection of animal life.

#### Murine inflammatory response to airway infection with alginate-embedded P. aeruginosa

##### Immobilization of bacteria in seaweed alginate beads

Immobilization of *P. aeruginosa* in seaweed alginate beads was performed as previously described (Pedersen *et al*., 1990). In brief, the bacteria were isolated from 100 ml of overnight culture grown in filtered oxbroth LB by centrifugation (12 000 *g*, 10 min, 4°C) and resuspension of the pellet in 5 ml sterile serum bouillon LB. One millilitre of bacterial suspension was mixed with 9 ml of sterile seaweed alginate (Protanal 10/60; Protan, Drammen, Norway). The mixture was forced with air through a channel into a solution of 0.1 M CaCl_2_ in 0.1 M Tris/HCl buffer (pH 7.0). The content of embedded bacteria in terms of cfu was estimated by serial dilutions and the challenge suspension of *P. aeruginosa* was adjusted to yield 8 × 10^4^ cfu ml^−1^.

##### Murine airway infections

Ten- to twelve-week-old female C3H/HeN mice were anaesthetized by subcutaneous injection of 0.2 ml Hyp/Mid [2.5 ml mg^−1^ Hypnorm (Janssen, Birkerød, Denmark) and 1.25 mg ml^−1^ Midazolam (Roche, Basel, Switzerland)] in sterile water prior to challenge. The trachea was exposed and penetrated with an 18G needle. Intratracheal challenge was performed by installing 40 μl (8 × 10^4^ cfu ml^−1^) of alginate-embedded bacteria in the left lung ∼11 mm from the tracheal penetration site with a bead-tipped curved needle (Moser *et al*., 1997). The incision was sutured and healed without any complications. The mice were sacrificed on day 4 by intraperitoneal injection of 0.05 ml 20% pentobarbital (KVL, Copenhagen, Denmark).

##### Blood leucocytes

The thoracic cavity was exposed and blood was collected from the heart in heparinized syringes and immediately put on ice.

##### Bronchoalveolar lavage

The trachea was exposed and canulated with a size 22G catheter (OPTIVA* 2, Johnson and Johnson Medical, Brussels, Belgium). A lavage was performed six times by repeated flushing with 1.5 ml ice-cold PBS and the BAL fluid was stored on ice.

##### Measurement of leukocytes from blood and BAL-fluid

The fraction of PMNs was estimated by adding 75 μl peripheral blood or 100 μl BAL-fluid to 4 ml cold 0.87% (w/v) NH_4_Cl buffer in order to lyse the erythrocytes. The cell pellet obtained after centrifugation was washed in PBS before adding the antibodies listed in Table S3. The cells were incubated on ice in the dark for 30 min and washed once in cold PBS. The pellet was resuspended in 200 μl before flow cytometry. The concentration of PMNs was measured by TruCount (340334, Becton Dickinson). Briefly, 200 μl BAL fluid or 50 μl blood was added to a TruCount Tube. Cells were fixed and the DNA was stained by adding 450 μl of 10% (v/v) FACS lysing solution (349202, Becton Dickinson) and 100 μg ml^−1^ propidium iodide (P-4170, Sigma) in MilliQ water. The samples were kept on ice in the dark for at least 10 min before analysing by flow cytometry.

The endobronchial content of PMNs was estimated by multiplying the concentration of PMNs with the volume of recovered BAL fluid.

##### Flow cytometry

The samples were analysed using a FACSort (Becton Dickinson) equipped with a 15 mW argon-ion laser tuned at 488 nm for excitation and a red diode laser for excitation at 635 nm. Light scatter and logarithmically amplified fluorescence parameters from at least 10 000 events, when possible, were recorded in list mode after gating on light scatter to avoid debris, cell aggregates, and bacteria. The instrument was calibrated using Calibrite (Becton Dickinson). Leucocytes were identified according to their morphology, by their expression of CD45, and their content of DNA.
