# Supplementary material for: Multiple roles of Pseudomonas aeruginosa TBCF10839 PilY1 in motility, transport and infection
Source: Mol Microbiol. 2008 Dec 10;71(3):730–47. doi: 10.1111/j.1365-2958.2008.06559.x (PMC2680260; doi:10.1111/j.1365-2958.2008.06559.x)
Supplement: Supplementary file 1 [file mmi0071-0730-SD1.pdf]

Multiple roles of *Pseudomonas aeruginosa* TBCF10839 PilY1 in motility, transport and infection

Yu-Sing Tammy Bohn[1¶], Gudrun Brandes[2], Elza Rakhimova[1], Sonja Horatzek[1], Prabhakar Salunkhe[1], Antje Munder[1], Andrea van Barneveld[1], Doris Jordan[1,3], Florian Bredenbruch[4$], Susanne Häußler[4], Kathrin Riedel[5], Leo Eberl[5], Peter Østrup Jensen[6], Thomas Bjarnsholt[6,7], Claus Moser[6], Niels Hoiby[6], Burkhard Tümmler[1], Lutz Wiehlmann[1]

[1]Klinische Forschergruppe, OE 6710, [2]Abteilung Zellbiologie, OE 4130, and Betriebseinheit Elektronenmikroskopie-Labor, OE 8840, [3]Abteilung für Medizinische Mikrobiologie und Krankenhaushygiene, OE 5210, Medizinische Hochschule Hannover, Carl-Neuberg-Str. 1, D-30625 Hannover, Germany;

[4]Abteilung für Zellbiologie und Immunologie, Helmholtz Institut für Infektionsforschung, Inhoffenstr. 7, D-38124 Braunschweig, Germany;

[5]Abteilung für Mikrobiologie, Institut für Pflanzenbiologie, Universität Zürich, Winterthurerstrasse 190, CH-8057 Zürich, Switzerland;

[6]Department of Clinical Microbiology, Rigshospitalet, afsnit 9301, Department of Clinical Microbiology, Juliane Maries Vej 22, DK-2100 Copenhagen, Denmark;

[7]BioScience and Technology BioCentrum-DTU, Building 227, Technical University of Denmark, DK-2800 Lyngby, Denmark

[¶] present address: Laboratoire de Biochimie et Biophysique des Systèmes Intégrés, iRTSV/CEA-Grenoble, 17 rue des Martyrs, F-38054 Grenoble, France

[$] present address: QIAGEN GmbH, QIAGEN Str. 1, D-40724 Hilden, Germany

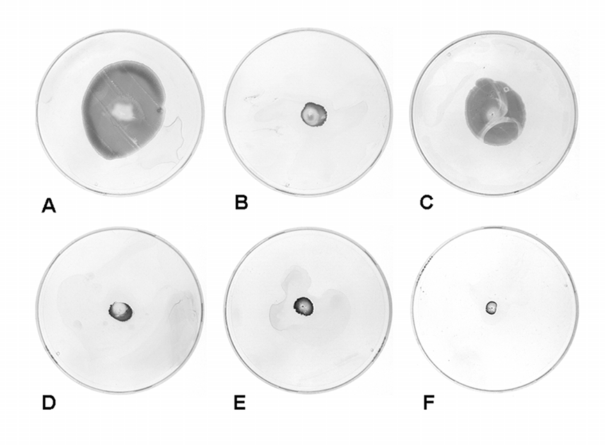

Figure S1.

Twitching motility of *P. aeruginosa* strains. In contrast to *P. aeruginosa* PAO1 (A) the non-piliated strain TBCF10839 (B) cannot twitch due to an intragenic deletion in *pilQ* (Chang *et al.,* 2007) which can be reverted by complementation with the PAO1-derived *pilQ* gene in trans (C). Likewise, the PilY1^{-} transposon mutant of TBCF10839 (25C8) (D) is non-motile and complementation with TB *pilY1* (E) or PAO1 *pilQ* (F) *in trans* did not restore the twitching phenotype.

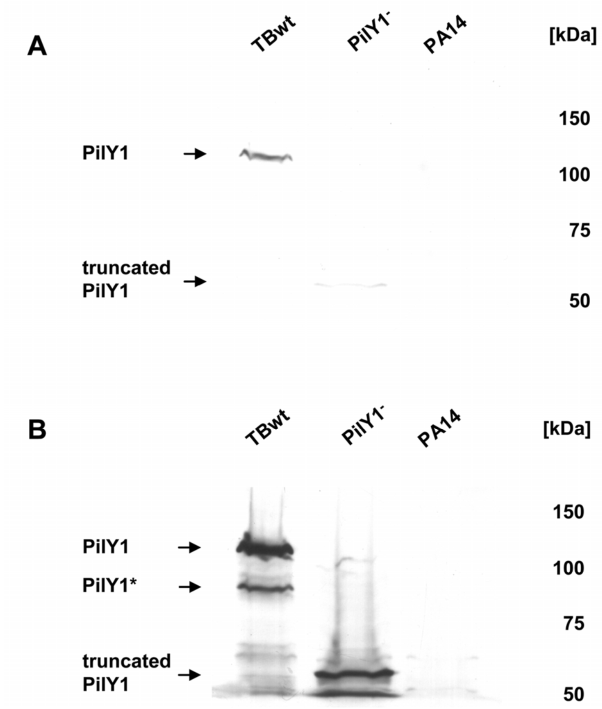

Figure S2.

Specificity of the anti-PilY1(TBCF10839) antibody to detect PilY1 isoforms. Anti- PilY1(TBCF10839) immunoblot of 6% PAGE-separated cell lysates of strains TBCF10839 (left), TBCF10839 *pilY1*::Tn*5* (mutant 25C8) (middle) and PA14 (right). The polyclonal antibody recognized an immunoreative band of predicted size for PilY1 (126 kD) for the wild-type strain and a truncated isoform (~ 59 kD) for transposon mutant 25C8 (**A**) . PilY1 exhibits substantial amino acid sequence diversity (see Figure 14). The peptide epitopes used for immunization are not present in the primary amino acid sequence of PilY1 of strain PA14. Correspondingly PilY1 of PA14 was not recognized by the polyclonal antiPilY1(PAO1/TBCF10839) antibodies. A shorter isoform PilY1* not visible by exposure for 1 min (**A)** became apparent in the TBCF10839 lane by exposure with the ECL reagent for 20 min (**B**). Please note that the PA14 lane in (**B**) still did not show any non-specific immunoreactive signal.

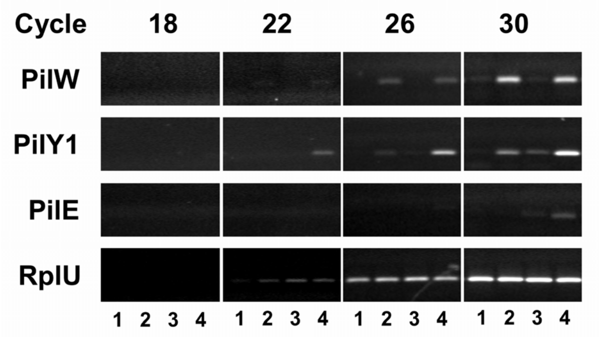

Figure S3.

Quantitation of PilW, PilY1, PilE and RplU mRNA transcripts in strains TBCF10839 (1), TBCF10839 pilY1::Tn5 mutant 25C8 (2), PA14 (3) and PA14 pilY1 mutant ID 25263 (4) by RT/PCR kinetics. cDNA was synthesized from 500 ng RNA by RT as described in Experimental Procedures. A 0.25 % aliquot of the RT reaction mixture was subjected to PCR. Aliquots were withdrawn at the indicated reaction cycles. The gel-separated PCR products were stained with ethidium bromide. RplU was chosen as the constitutively expressed housekeeping gene.

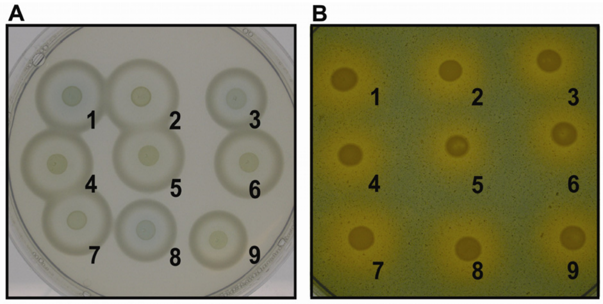

Figure S4

Proteolytic activity on casein agar (**A**) and siderophore activity on chromazurol S agar (CAS agar) (**B**) by *P. aeruginosa* strains: 1, TBCF10839; 2, PAO1; 3, PA14; 4, TBCF10839 *pilY1*::Tn*5* (25C8); 5, TBCF10839 *pilY1*::Tn*5* (10CB5); 6, TBCF10839 *pilW*::Tn*5*; 7, TBCF10839 *pilY1*::Tn*5* (25C8) complemented with pME6010::TB*pilY1*; 8, TBCF10839 *pilY1*::Tn*5* (10CB5) complemented with pME6010::TB*pilY1*; 9, TBCF10839 *pilW*::Tn*5* complemented with ME6010::TB*pilW*. All nine strains were proficient in the secretion of proteases and siderophores.

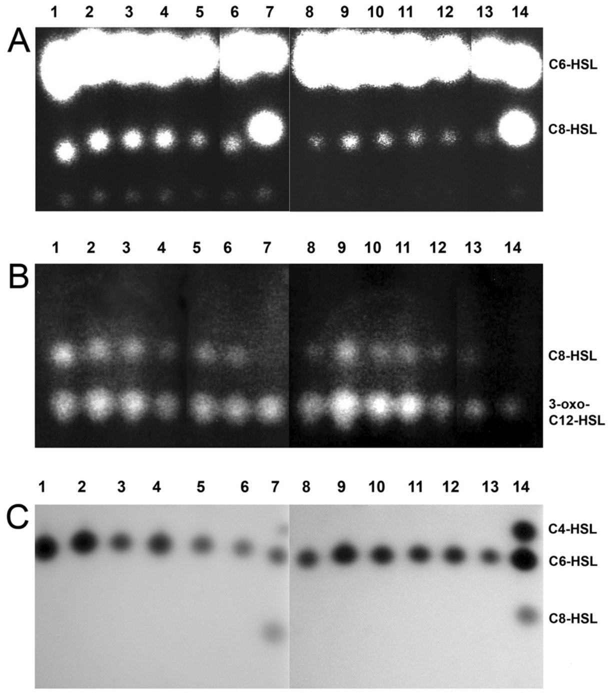

Figure S5.

Secretion of *N*- acylhomoserine lactones (AHLs) by *P. aeruginosa* strains. TLC analysis of supernatants of strains 1, *P. aeruginosa* PAO1; 2, TBCF10839; 3, TBCF10839 complemented with pME6010 (vector control); 4, TBCF10839 *pilW*::Tn*5*; 5, TBCF10839 *pilW*::Tn*5* complemented with pME6010 (vector control); 6, TBCF10839 *pilW*::Tn*5* complemented with pME6010::TB*pilW*; 7, standards; 8, PA14; 9, PA14 *MAR2xT7 pilY1* mutant ID25563; 10, TBCF10839 *pilY1*::Tn*5* (mutant 25C8); 11, TBCF10839 *pilY1*::Tn*5* (mutant 25C8) complemented with pME6010 (vector control); 12, TBCF10839 *pilY1*::Tn*5* (mutant 25C8) complemented with pME6010::TB*pilY1*; 13, TBCF10839 *pilY1*::Tn*5* (mutant 10CB5); 14, standards (C4-HSL, *N*-butanoyl homoserine lactone; C6-HSL, *N*-hexanoyl homoserine lactone; C8-HSL, *N*-octanoyl homoserine lactone; 3-oxo-C12-HSL *N*-(3-oxododecanoyl) homoserine lactone). AHL molecules were extracted from spent culture supernatants of the strains, separated by TLC and visualized by overlaying the TLC plates with soft agar seeded with the *luxAB* based AHL biosensor *Escherichia coli* MT102 (pSB403) (Winson *et al.,* 1998), biosensor *Chromobacterium violaceum* CV026 (McClean *et al*., 1997) or biosensor *Pseudomonas putida* F117 (pKR-C12) (Steidle *et al.,* 2001). The biosensors respond to different types of AHL molecules with differential sensitivity. Bioluminescent spots indicating AHLs were detected by exposure of a X-ray film. On the basis of their mobilities (R_f-values) and by including appropriate reference compounds a tentative identification of AHLs present in the culture extracts was possible. All investigated strains secreted comparable amounts of AHLs.

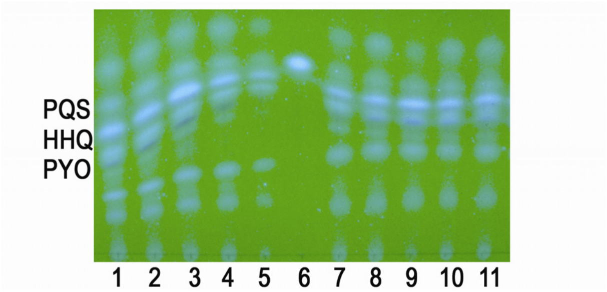

Figure S6.
Thin layer chromatogram of HAQ metabolites from extracts of whole cultures grown to OD_{578} = 2.5 (Lanes 1-5) or grown for 15 hours (lanes 7-11) in LB broth of *P. aeruginosa* strains PA14 *pilY1* mutants ID25263 (lanes 1, 11) and ID 25563 (lanes 2, 10); PA14 (lanes 3, 9); TBCF10839 (lanes 4, 8); TBCF10839 *pilY1*::Tn*5* (25C8) (lanes 5, 7). PQS was applied as standard in lane 6.

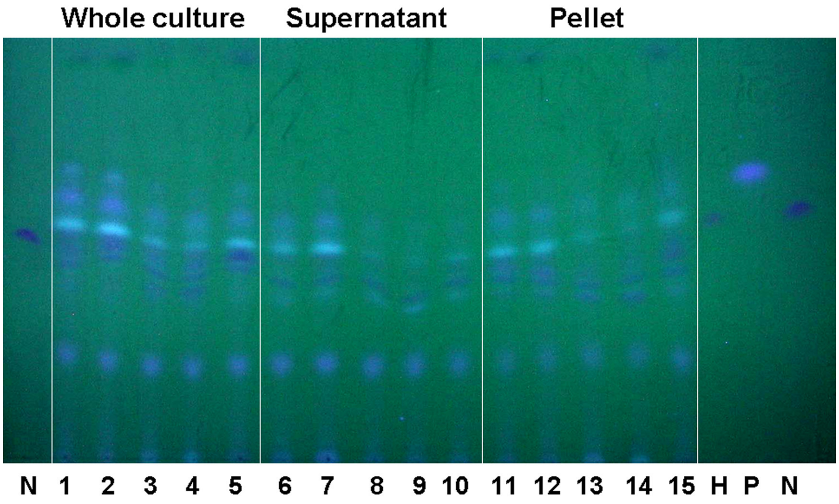

Figure S7.

Thin layer chromatogram of HAQ metabolites from extracts of whole culture, supernatant and pellet fractions of *P. aeruginosa* strains TBCF10839 (lanes 1, 6, 11), TBCF10839 carrying plasmid pME6010 (lanes 2, 7, 12), TBCF10839 *pilY1*::Tn*5* (25C8) (lanes 3, 8, 13), TBCF10839 *pilY1*::Tn*5* (25C8) carrying plasmid pME6010 (lanes 4, 9, 14) and TBCF10839 *pilY1*::Tn*5* (25C8) complemented with pME6010::TB*pilY1* (lanes 5, 10, 15). Inactivation of *pilY1* reduced the total concentration of HAQs only small portions of which were present in the extracellular space. The chemically synthesized HAQs 4-hydroxy-2-heptylquinoline (H), 3,4-dihydroxy-2-heptylquinoline (PQS) and 4-hydroxy-2-heptylquinoline N-oxide (N) were included as standards.

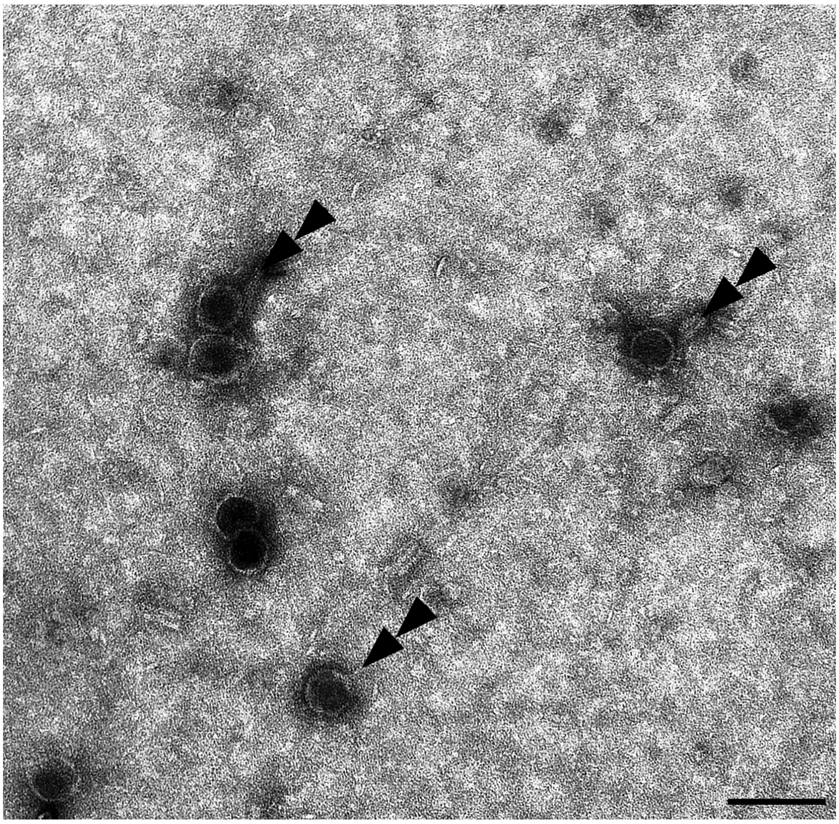

Figure S8.

Electron micrograph of negatively stained phage F116. *P. aeruginosa* PAO1 was transduced with phage F116, a temperate, pilus-specific, generalized transducing phage belonging to the Podoviridae virus family (Byrne and Kropinski, 2005). The double arrowhead points to the phage (bar: 100 nm; original magnification 50,000). Colonies were processed for electron microscopy according to the same protocol that was used to visualize extracellular PilY1-immunoreactive structures (see Figure 9 and 'Experimental Procedures'). Please note that no phage was detected in the analysis of *P. aeruginosa* PAO1, TBCF10839 and its isogenic mutants and strains complemented *in trans* (Figure 9).

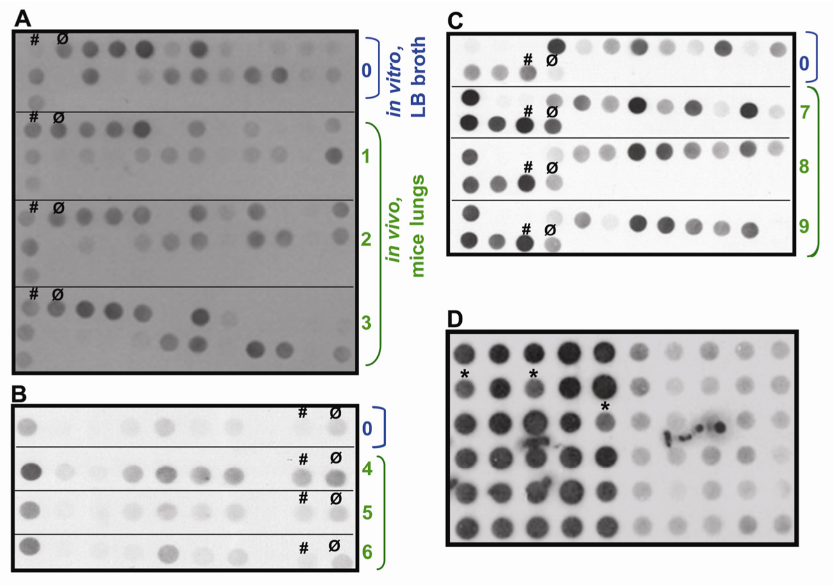

Figure S9.

A, B, C. Dot blot hybridization of oligonucleotide signature tags to determine the survival of individual P. aeruginosa TBCF10839 mutants in competition experiments (A, B, C) in murine lungs in vivo. The total pool of isogenic mutants was tested in different combinations (Rakhimova et al., 2008). DIG-labeled, HindIII-digested signal sequences isolated from bacteria cultured in vitro in LB broth (0) or from bacteria recovered from infected murine lungs were hybridized onto dot blots of the signal sequences of the pTnModOGm SigTag. The arabic numerals indicate different mice and the capital letters A, B, C indicate different combinations of mutants. #, hybridization signal of the signature tag of the TBCF10839 pilY1::Tn5 25C8 mutant; Ø, hybridization signal of the signature tag of the TBCF10839 pilW::Tn5 14D1 mutant.

D. Preselection of oligonucleotide signature tags (Control experiment prior to the construction of the STM library). A panel of 30 oligonucleotide tags (Wiehlmann et al., 2007b) was hybridized onto a dot blot on which the set of 30 complementary sequences had been immobilized on the left five rows and a set of 30 related sequences with a single mismatch each had been immobilized on the right five rows. Tags with specific and strong hybridization signals of comparable intensity were chosen for the construction of the STM library. Tags were sorted out that showed cross-hybridization or yielded only weak hybridization signals (marked by an asterisk * in panel D). Hence, the different hybridization signals shown in panels A-C do not reflect differences in the affinity of oligonucleotide tags towards their complementary sequence. Instead, the intensity of a hybridization signal is the read-out of a STM experiment (Mazurkiewicz et al., 2006) to detect the relative abundance of individual STM mutants in the tested habitats (LB broth and murine airways in this case).

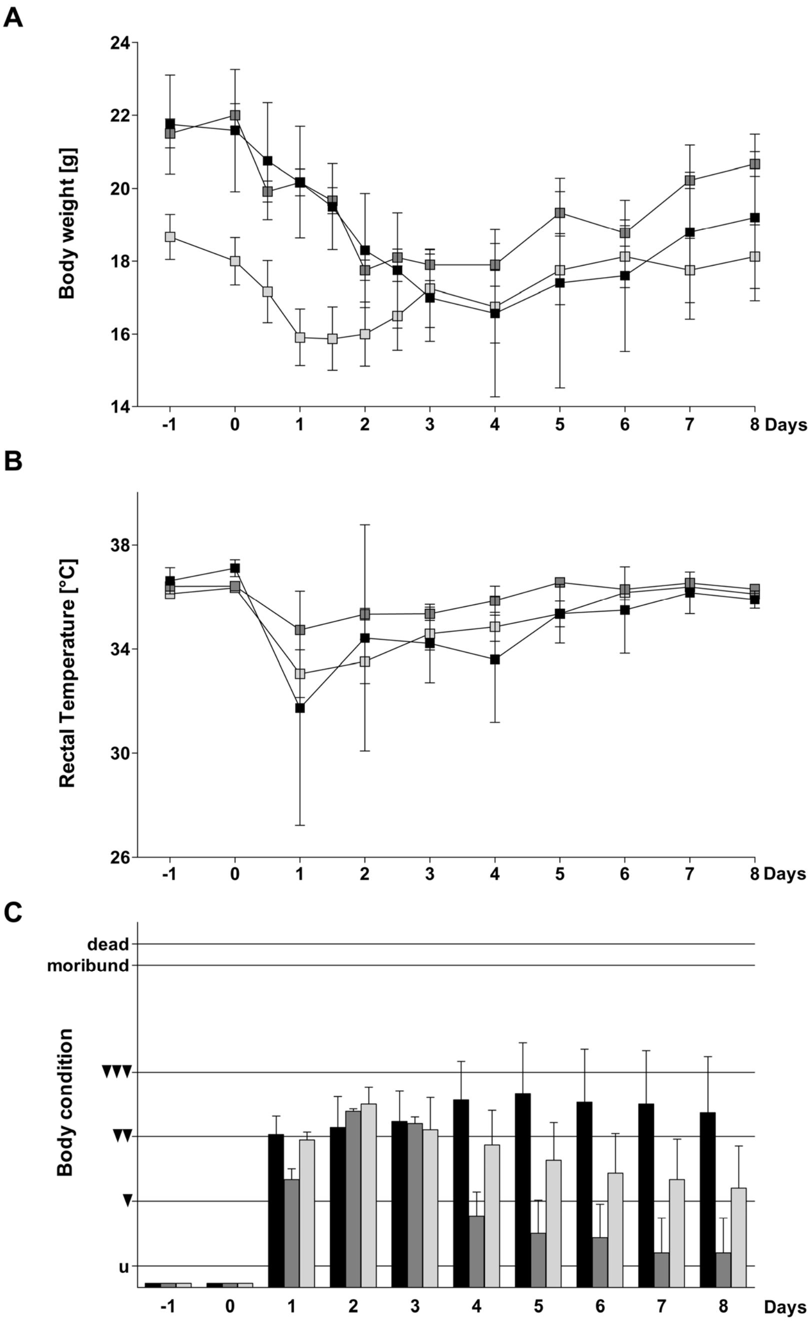

Figure S10.

Acute murine airway infection model: Body weight, rectal temperature, body condition score.

7.5 x 10^6 cfu of *P. aeruginosa* TBCF10839 (black); B, TBCF10839 *pilY1*::Tn*5* (25C8) (grey); C, TBCF10839 *pilW*::Tn*5* (light grey) were instilled intratracheally into murine airways (Munder *et al.,* 2002). To characterize the course of the bacterial infection, the body weight (**A**) and rectal temperature of the mice (**B**) were measured daily and their body condition (**C**) was evaluated for the parameters vocalization, piloerection, attitude, locomotion, breathing, curiosity, nasal secretion, grooming and dehydration (Munder *et al.,* 2002). Dysfunctions in single behavioral parameters were assessed by zero, one or two points, respectively. The body condition of the mice was determined by adding the points resulting in the following score: untroubled 0–1 (u); slightly troubled 2–4 (▼); moderately troubled 5–7 (▼▼); profoundly troubled 8–10 (▼▼▼); moribund ≥ 11; death ≥ 16.

A. Body weight. Infection with TBCF10839 wild type caused the most distinct loss of body weight of mice. The majority of the animals in this group started late to return to normal weight gain by day 5 p.i.. The animals infected with TBCF10839 *pilY1*::Tn*5* and TBCF10839 *pilW*::Tn*5* also showed weight loss but recovered significantly earlier around day 2 p.i..

B. Rectal temperature. Mice infected with the wild type strain had the most pronounced decrease in temperature on day 1 p.i.. Thereafter the time course of the rectal temperature of the animals was similar.

C. Body condition. The columns in the panel display the time course of the body condition score (mean values±SE, *n*=10). Mice infected with TBCF10839 wild type showed a strongly higher disturbance over the infection time than mice infected with either TBCF10839 *pilY1*::Tn*5* or TBCF10839 *pilW*::Tn*5*, respectively.

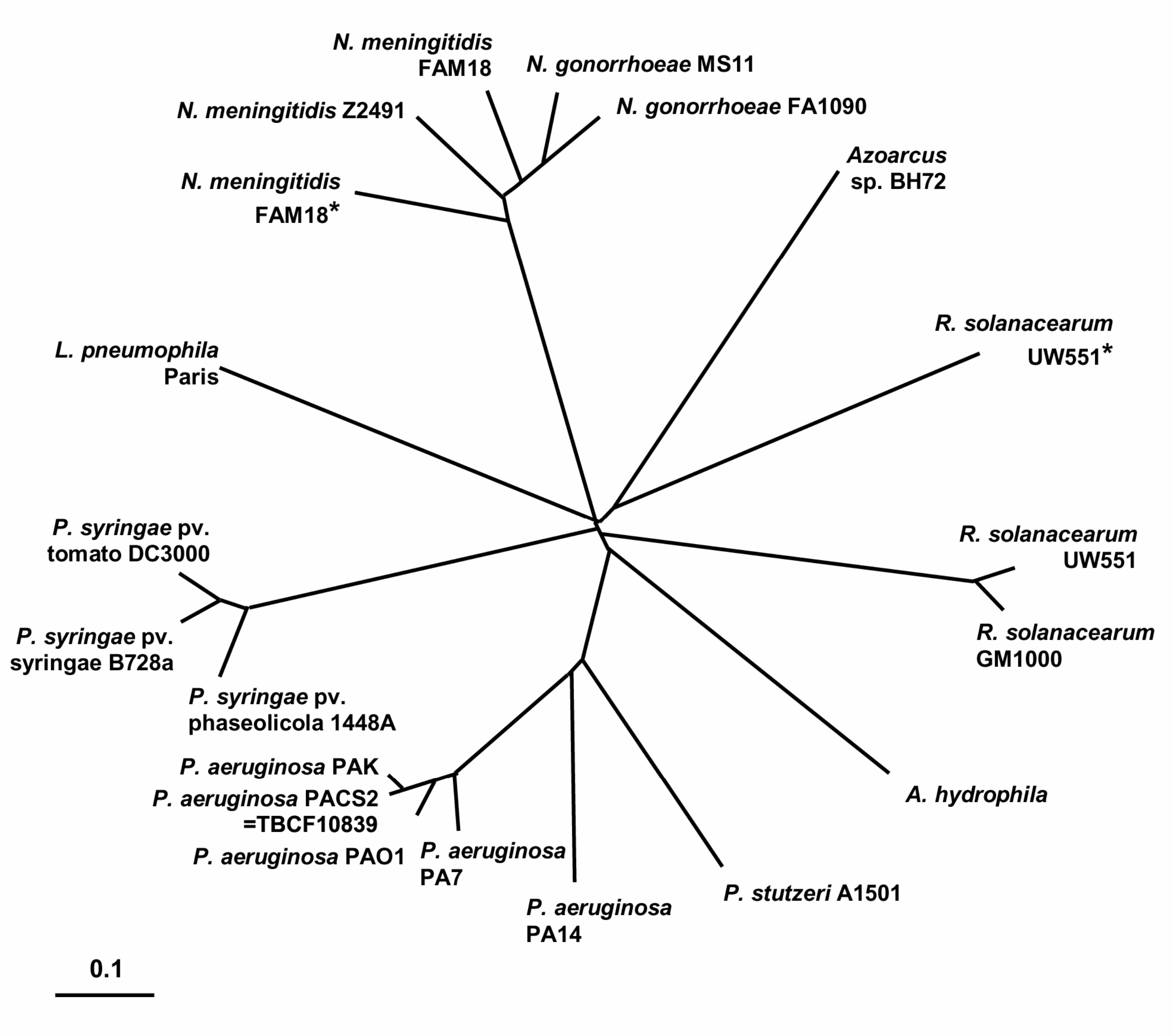

Figure S11.

Dendrogram of *P. aeruginosa* TBCF10839 PilY1 related proteins. The Genbank database (February 1, 2008) was searched for *pilY1* homologues in the 67 bacterial strains with conserved T4P core genes. Sequences were aligned by CLUSTALX. The unrooted neighbour-joining tree was drawn for the PilY1 homologues with the highest similarity by excluding the positions with gaps. Strains with two PilY1 homologues are indicated with an asterisk. The highly similar PilY1-related proteins of *Legionella pneumophila* strains are represented by the sequence of strain L. pneumophila Paris. Two major subtypes can be differentiated among the *P. aeruginosa* PilY1 proteins of completely sequenced strains, one type represented by strains PAO1, PACS2 and PA7, the other type represented by strains PA14 and C3719. Please note that the amino acid sequences of the PilY1 proteins of *P. aeruginosa* strains PACS2 and TBCF10839 are identical.

Table S1.
PAO1 Gene Chip mRNA expression analysis of *P. aeruginosa* strains TBCF10839 and TBCF10839 *pilY1*::Tn*5* grown in LB broth to early stationary phase

Gene locus	Annotation: Function / Name	Functional class	Classification of signal in TBCF10839 (left) and TBCF10839 pilY1::Tn5 (right) P/M/A*	Normalized signal of mRNA expression in TBCF10839	Differential expression in the TBCF10839 pilY1::Tn5 mutant	
					x-fold down-regulated wt/mutant	x-fold up-regulated mutant/wt
Intergenic region	between PA1934 and PA1935	2068728-2069490, (-) strand	002-011	6.5	0.1	8.1
Intergenic region	between PA1372 and PA1373	1427453-1428080, (+) strand	200-002	71.2	3.8	0.3
Intergenic region	between PA4581 and PA4582	5086695-5087407, (+) strand	011-002	40.3	4.0	0.3
M29695	6 -N-acetyltransferase (AAC (6)-II) gene	antibiotic resistance (Gentamicin)	002-200	1.7	0.0	947.2
PA0173	probable methylesterase	Adaptation, protection; Chemotaxis	200-002	218.8	3.7	0.3
PA0233	probable transcriptional regulator	Transcriptional regulators	200-002	56.5	3.6	0.3
PA0243	probable transcriptional regulator	Transcriptional regulators	200-002	84.9	4.9	0.2
PA0350	folA; dihydrofolate reductase	Biosynthesis of cofactors, prosthetic groups and carriers	200-002	61.2	3.6	0.3
PA0361	probable gamma-glutamyltranspeptidase precursor	Amino acid biosynthesis and metabolism	200-002	162.6	5.5	0.2
PA0600	probable two-component sensor	Two-component regulatory systems	101-002	24.6	7.4	0.1
PA0605	probable permease of ABC transporter	Membrane proteins; Transport of small molecules	200-002	70.8	3.3	0.3
PA0633	hypothetical protein	Related to phage, transposon, or plasmid	002-011	2.7	0.0	20.2
PA0733	probable pseudouridylate synthase	Transcription, RNA processing and degradation	110-101	25.3	0.1	8.3
PA0737	hypothetical protein	Hypothetical, unclassified, unknown	020-002	26.2	5.7	0.2
PA0758	hypothetical protein	Hypothetical, unclassified, unknown	200-200	94.0	0.1	9.6
PA0799	probable helicase	Putative enzymes	011-002	31.4	3.2	0.3
PA0882	hypothetical protein	Putative enzymes	002-011	3.7	0.1	12.1
PA0937	conserved hypothetical protein	Hypothetical, unclassified, unknown	200-002	193.1	3.8	0.3
PA1006	conserved hypothetical protein	Hypothetical, unclassified, unknown	101-002	35.5	4.7	0.2
PA1234	hypothetical protein	Hypothetical, unclassified, unknown	200-002	67.5	3.5	0.3
PA1301	probable transmembrane sensor	Membrane proteins; Transcriptional regulators	002-110	10.4	0.1	7.8
PA1355	hypothetical protein	Hypothetical, unclassified, unknown	110-002	20.6	3.3	0.3
PA1386	probable ATP-binding component of ABC transporter	Transport of small molecules	101-002	16.6	3.0	0.3
PA1672	hypothetical protein	Hypothetical, unclassified, unknown	200-002	41.0	6.3	0.2

PA1693	pscR; translocation protein in type III secretion	Protein secretion	011-002	24.8	4.6	0.2
PA1695	pscP; translocation protein in type III secretion	Protein secretion	200-002	68.4	7.2	0.1
PA1780	nirD; assimilatory nitrite reductase small subunit	Central intermediary metabolism	002-011	1.8	0.1	17.7
PA2082	probable transcriptional regulator	Transcriptional regulators	110-002	39.2	3.9	0.3
PA2143	hypothetical protein	Hypothetical, unclassified, unknown	200-002	150.9	3.6	0.3
PA2158	probable alcohol dehydrogenase (Zn-dependent)	Putative enzymes	200-002	90.6	3.6	0.3
PA2183	hypothetical protein	Hypothetical, unclassified, unknown	011-002	26.0	4.2	0.2
PA2272	pbpC; penicillin-binding protein 3A	Cell wall / LPS / capsule	200-002	46.0	3.0	0.3
PA2312	probable transcriptional regulator	Transcriptional regulators	002-200	8.7	0.1	8.4
PA2321	gluconokinase	Carbon compound catabolism; Energy metabolism	002-020	16.0	0.1	7.2
PA2417	probable transcriptional regulator	Transcriptional regulators	011-002	32.8	3.6	0.3
PA2449	probable transcriptional regulator	Transcriptional regulators	200-002	31.2	9.5	0.1
PA2577	probable transcriptional regulator	Transcriptional regulators; Membrane proteins	200-002	118.0	4.4	0.2
PA2727	hypothetical protein	Hypothetical, unclassified, unknown	200-002	84.5	4.5	0.2
PA3019	probable ATP-binding component of ABC transporter	Transport of small molecules	110-002	34.8	4.5	0.2
PA3238	hypothetical protein	Hypothetical, unclassified, unknown	200-002	124.2	6.9	0.1
PA3242	probable lauroyl acyltransferase	Cell wall / LPS / capsule	101-002	36.3	6.9	0.1
PA3350	hypothetical protein	Hypothetical, unclassified, unknown	200-002	136.2	3.6	0.3
PA3414	hypothetical protein	Hypothetical, unclassified, unknown	200-002	47.1	4.0	0.2
PA3427	probable short-chain dehydrogenases	Putative enzymes	110-002	66.0	3.5	0.3
PA3488	hypothetical protein	Hypothetical, unclassified, unknown	002-110	8.1	0.1	9.1
PA3499	hypothetical protein	Hypothetical, unclassified, unknown	002-011	9.2	0.1	8.3
PA3578	conserved hypothetical protein	Hypothetical, unclassified, unknown	200-200	51.1	0.1	10.5
PA3733	hypothetical protein	Hypothetical, unclassified, unknown	200-002	62.4	5.9	0.2
PA3814	iscS; L-cysteine desulfurase (pyridoxal phosphate-dependent)	Amino acid biosynthesis and metabolism; Biosynthesis of cofactors	200-200	1338.7	4.2	0.2
PA3815	conserved hypothetical protein	Hypothetical, unclassified, unknown	200-011	360.4	3.5	0.3
PA3830	probable transcriptional regulator	Transcriptional regulators	011-002	30.9	3.4	0.3
PA3905	hypothetical protein	Hypothetical, unclassified, unknown	200-002	89.0	6.1	0.2
PA3963	probable transporter	Membrane proteins; Transport of small molecules	011-002	36.2	4.0	0.2
PA4171	probable protease	Putative enzymes	200-110	265.5	4.7	0.2
PA4307	pctC; chemotactic transducer PctC	Adaptation, protection; Chemotaxis	110-002	78.0	3.3	0.3
PA4326	hypothetical protein	Hypothetical, unclassified, unknown	200-002	148.1	4.9	0.2
PA4401	probable glutathione S-transferase	Putative enzymes	200-002	138.7	3.2	0.3
PA4550	fimU; type 4 fimbrial biogenesis protein FimU	Motility & Attachment	200-200	78.9	0.0	21.7
PA4551	pilV; type 4 fimbrial biogenesis protein PilV	Motility & Attachment	200-200	132.7	0.1	15.7
PA4552	pilW; type 4 fimbrial biogenesis protein PilW	Motility & Attachment	200-200	100.0	0.1	19.5
PA4553	pilX; type 4 fimbrial biogenesis protein PilX	Motility & Attachment	200-200	67.0	0.0	26.8
PA4554	pilY1; type 4 fimbrial biogenesis protein PilY1	Motility & Attachment	200-200	89.5	0.1	7.4
PA4679	hypothetical protein	Hypothetical, unclassified, unknown	101-002	49.9	6.5	0.2
PA4780	conserved hypothetical protein	Hypothetical, unclassified, unknown	200-200	182.5	0.2	6.0
PA4960	probable phosphoserine phosphatase	Amino acid biosynthesis and metabolism	200-002	110.3	10.8	0.1
PA5031	probable short chain dehydrogenase	Putative enzymes	101-101	18.7	0.2	6.2

PA5138	hypothetical protein	Hypothetical, unclassified, unknown	200-002	33.6	4.1	0.2
PA5275	conserved hypothetical protein	Hypothetical, unclassified, unknown	200-002	63.1	8.2	0.1
PA5284	hypothetical protein	Hypothetical, unclassified, unknown	011-002	12.2	4.2	0.2
PA5462	hypothetical protein	Hypothetical, unclassified, unknown	200-002	34.6	3.7	0.3
PA5467	hypothetical protein	Hypothetical, unclassified, unknown	110-002	18.7	3.5	0.3
PA5472	hypothetical protein	Hypothetical, unclassified, unknown	110-002	75.2	3.8	0.3
PA5511	probable two-component response regulator	Transcriptional regulators; Two-component regulatory systems	110-002	61.4	4.3	0.2
PA5524	probable short-chain dehydrogenase	Putative enzymes	200-002	35.1	5.4	0.2
PA5533	hypothetical protein	Hypothetical, unclassified, unknown	110-002	27.7	3.4	0.3
tRNA(Tyr)	tRNA_Tyrosine	4785788-4785872 (-) strand	200-002	9.5	47.5	0.0

*no. of chips with the hybridization signal of the respective gene classified present (P, 1st digit) / marginally expressed (M, 2nd digit) / absent (A, 3rd digit)

Table S2. Strains, plasmids and oligonucleotide primers.

**Strains**	**Genotype and/or source**	**Reference**
E. coli DH5α	F$^-$, φ80m80*1acZ*ΔM15, Δ(*lacYZA-arg*F), U169*, rec*A1*, end*A1*, hsd*R17*,* $(r_{k-}; m_{k+})$, *pho*A, *sup*E44, *λ*$^-$*, thi*-1*, gyr*A96, *rel*A1	Raleigh *et al*., 1997
P. aeruginosa TBCF10839	CF airways, serotype 4; pyocin type: 1h, phage lysotype: F8, M4, PS2, PS24, PS31, 352, 46b/2, 1214, Col21, F7, F10, PS21, PS73, no plasmids. Hexadecimal SNP genotype (Wiehlmann *et al*., 2007a): 3C52.	Tümmler *et al*., 1991
P. aeruginosa PA14	Human clinical isolate; hexadecimal SNP genotype: D421	Rahme *et al*., 1995
**Plasmids**	**Genotype and/or source**	
pME6010	Shuttle vector for Gram-negative bacteria; Tc^r	Heeb *et al.,* 2000
pUCP20	*Escherichia-Pseudomonas* shuttle vector; Ap^r	Raleigh *et al*., 1997
pME6010::PA*pilQ*	pME6010 containing the BglI/EcoRI PCR product bearing the PAO1 *pilQ* gene	Chang *et al*., 2007
pME6010::TB*pilY1*	pME6010 containing the BglI/EcoRI PCR product bearing the TBCF10839 *pilY1* gene	This study
pME6010::TB*pilW*	pME6010 carrying the KpnI/EcoRI PCR product bearing the TBCF10839 *pilW* gene	This study
pUCP20::TB*mvfR*	pUCP20 carrying the HindIII/SacI PCR product bearing the TBCF10839 *mvfR* (*pqsR*) gene	Rakhimova *et al*., 2008
**Primers**	**Sequence**	**Reference**
5’pilW_KpnI<br>3’pilW_EcoRI	GCCGGTACCCGACTTCTTCAAGGCCAAGG<br>GCGAATTCCGCGCTGTTGTGCAGGGAAGT	This study
5’pilY1_Bgl<br>3’pilY1_EcoRI	CGGAGATCTGGAACAACCTGCCCATTCCC<br>GCCGAATTCGAAGGTCTGGGGATCTTCGG	This study
5’mvfR_HindIII<br>3’mvfR_SacI	GGATAAGCTTACACCTGAAGGCGCAACAGC<br>CTAGAGCTCCGGAAGGTTTCGACTGCCTG	Rakhimova *et al*., 2008
5’rplU (RT/PCR)<br>3’rplU	GTACGCAGTGATTGTTACCGG<br>ATGTGGTGCTTACGACGGCG	This study
5’TB.pilW (RT/PCR)<br>3’TB.pilW	CCTGATTCTCGGGATTACCC<br>CAATAGGCGGTTTTCTGCTG	This study
5’TB.pilY1 (RT/PCR)<br>3’TB.pilY1	AATCGGTACTCCACCAGATCG<br>GGCATAAGCCATACTGCCCG	This study
5’TB.pilE(RT/PCR)	ACTACGTGATCCGCTCCAAC	This study

3'TB.pilE	GGTCAGGGTATAGGTGGTGC	
5'PA14.pilW (RT/PCR)<br>3'PA14.pilW	CTTGTTGATGATCACGCTCC<br>GCCGTTTGACATTGCTATCG	This study
5'PA14.pilY1 (RT/PCR)<br>3'PA14.pilY1	ATGATCCACCAGATTACCCGC<br>GTAAGCCCAGGCCATACTGC	This study
5'PA14.pilE (RT/PCR)<br>3'PA14.pilE	AAGGACAGGCATTACTCAGC<br>TAACCTCCGTCGTTGGCTAC	This study
5'PA14::MAR2xT7 (*pilY1a*)<br>3'PA14::MAR2xT7 (*pilY1a*)	CGGATTTCCGAGCGCAACAC<br>CCGAATGTGGAGTTCGCCTC	This study, verification of transposon insertion
5'PA14::MAR2xT7 (*pilY1b*)<br>3'PA14::MAR2xT7 (*pilY1b*)	GACTGGAGCCAGCGCATGATC<br>CGTTCCGACCGTTCCAGATAC	This study, verification of transposon insertion
5'P1<br>5'P2	GTACCCCACTAGTCCCAAGC<br>GTACCTCCACTCACCCAAGC	Rakhimova *et al*., 2008
5' Y-linker<br>5' Tn*5*	CTGCTCGAGCTCAAGCTTCG<br>TGCGTTCGGTCAAGGTTCTGG	Kwon and Ricke, 2000
5'Y1<br>5'Y2	TTTCTGCTCGAGCTCAAGCTTCGAACGATGTACGGGGACACATG<br>TGTCCCCGTACATCGTTCGAACTACTCGTACCATCCACAT	Kwon and Ricke, 2000

Table S3. Monoclonal rat anti-mouse antibodies.

Antibody	Target	Conjugated Fluorochrome	Amount (μL)	Clone	Isotype	Source
Ly-6G	PMNs	PE	1	RB6-8C5	IgG_{2b}, κ	Pharmingen
F4/80	Monocytes	FITC	5	CI:A3-1	IgG_{2b}, κ	Serotec
CD11b	Mac-1	APC	5	M1/70	IgG_{2b}, κ	Pharmingen
CD45	Leukocytes	PerCP	5	30-F11	IgG_{2b}, κ	Pharmingen

FITC: Fluorescein isothiocyanate; PerCP: Peridinin chlorophyll A protein; PE: Phycoerythrin; APC: Allophycocyanin, Pharmingen, San Diego, CA, US; Serotec: Oslo, Norway.
